# Artificial Intelligence, Connected Care, and Enabling Digital Health Technologies in Rare Diseases With a Focus on Lysosomal Storage Disorders: Scoping Review

**DOI:** 10.2196/73612

**Published:** 2026-04-02

**Authors:** Alberta MC Spreafico, Luca Neri, Kim Angel, Cinzia Maria Bellettato, Maurizio Scarpa

**Affiliations:** 1 Health Innovation EVERSANA INTOUCH Salerno Italy; 2 Government, Health and Not for Profit Division SDA Bocconi School of Management Milano Italy; 3 Innovation for Global Health Institute Milan Italy; 4 WINFOCUS Milan Italy; 5 Iuliu Hațieganu University of Medicine and Pharmacy Cluj-Napoca Romania; 6 International MPS Network Vancouver, BC Canada; 7 MetabERN, Regional Coordinating Center for Rare Diseases Udine University Hospital Udine Italy

**Keywords:** digital health, artificial intelligence, connected care, telemedicine, rare diseases, lysosomal storage disorders, remote monitoring, registries

## Abstract

**Background:**

Rare diseases affect more than 300 million people globally, and only about 5% have approved therapies. Lysosomal storage disorders (LSDs) exemplify the diagnostic and long-term care complexity typical of rare diseases, and digital health technologies (DHTs), especially artificial intelligence (AI) and connected care (CC), are emerging tools to support LSD management.

**Objective:**

We aimed to map and synthesize peer-reviewed and gray literature from the past decade on DHTs relevant for LSD care, with a primary analytic focus on AI-enabled and CC solutions and a contextual mapping of other enabling DHTs. Evidence distribution was charted by population, care-journey phase, and outcome domains to identify gaps, methodological limitations, and timely priorities relevant for research, clinical practice implementation, and policies.

**Methods:**

We conducted a scoping review guided by a population, concept, context framework and operationalized through a Population, Intervention, Comparison, and Outcome (PICO)-informed data-charting structure to map study characteristics and reported outcomes, without causal or effectiveness assumptions and without risk-of-bias assessment. We searched PubMed, Google Scholar, and ClinicalTrials.gov for studies published between October 2015 and September 2024, complemented by AI-assisted discovery tools for citation extension. Reproducibility logs (search strings, run dates, filters, and stepwise counts) were maintained. Of 1751 records retrieved, 245 were included. Evidence was charted by LSD population, intervention class (AI, CC, and other enabling DHTs), outcome domains (patient, health care, and societal), and phase of the care journey.

**Results:**

Among 245 included records, 92.2% (226/245) were peer-reviewed, and 7.8% (19/245) were gray literature; no completed and published randomized controlled trials or LSD-specific systematic reviews were identified, with evidence dominated by small, single-center observational studies. Overall, 40 peer-reviewed records reported AI-driven DHTs, 89 reported CC DHTs, and 144 reported other enabling DHTs (some multilabeled). Evidence was concentrated mostly in Gaucher and Fabry diseases. Nearly half of the mapped literature focused on screening and diagnosis, with fewer records addressing treatment intensification, rehabilitation, and end-of-life care. Outcomes were predominantly health care delivery performance measures, with fewer patient and societal outcomes. AI applications mainly supported diagnostic decision support, phenotyping, monitoring, tracking, and risk stratification; CC commonly involved telemedicine, remote monitoring, and patient-engagement platforms; enabling DHTs included interoperable data systems, registries, and digital infrastructures.

**Conclusions:**

The evidence base is appreciable for a niche field and reflects growing interest in AI and CC for LSD care, but heterogeneity and methodological limitations preclude inferences on effectiveness or routine implementation. This evidence map highlights relatively stronger areas and gaps, providing a structured foundation to inform timely expert consensus-building and research prioritization. Key priorities include interoperable data infrastructures and data availability, prospective multicenter evaluations, transparent reporting of algorithms and workflows, and implementation-relevant outcomes to support safe, equitable, and scalable adoption aligned with evolving European Union and global rare-disease priorities.

## Introduction

### Rationale

Lysosomal storage disorders (LSDs) are a subset of rare diseases (RDs), resulting from inherited deficiencies in lysosomal enzymes or transporters. They affect approximately 1 in 7000 to 8000 newborns and encompass over 70 disorders. They exemplify rare, chronic, and multisystem diseases that require coordinated, longitudinal, multispecialty care [1-4]. The 2021 United Nations resolution on “Addressing the Challenges of Persons Living with a Rare Disease and their Families” [5], together with several policy statements from the European Union (EU) and the World Health Organization, recognizes RDs as a global policy priority and calls for coordinated action and strengthened care networks to advance equitable Universal Health Coverage by 2030 [3].

Across RDs, the diffusion of digital health technologies (DHTs) has accelerated in both research and care delivery, reflecting the need to enhance early and accurate diagnosis, reduce geographic barriers, improve inclusion for dispersed populations, and support longitudinal follow-up [6,7]. DHTs have also been increasingly adopted in RD clinical trials to enable remote assessments and decentralized research models [8]. In parallel, regulators and health systems are integrating digital health into health policies and health technology assessment (HTA) frameworks, with growing emphasis on clinical validation, transparency, and safety. These cross-cutting developments are especially relevant to LSDs, where chronic multisystem trajectories require coordinated multidisciplinary care, timely specialist input, and monitoring across the life course.

However, despite this rapid diffusion and growing policy attention, the landscape of DHT applications in LSD care remains poorly characterized. While the burden and care complexity of RDs, such as LSDs, are well established, the evolving contributions of artificial intelligence (AI) and connected care (CC), as distinct yet complementary classes of DHTs with the potential to enhance LSD management, have not been systematically mapped [3,5,9]. Over the past decade, AI applications (eg, pattern recognition for early diagnosis, predictive analytics, and data-driven decision support systems [DSS]) and CC solutions (eg, televisits, remote monitoring, and digital medical devices [DMD]) have been increasingly reported in LSD contexts. However, evidence is dispersed across heterogeneous study designs, gray literature, and emerging evaluations, which limits informed decision-making for clinicians, patient organizations, and policymakers. Prior reviews survey broader rare-disease contexts or single DHT classes, but none provide an LSD-specific evidence map specifying the studied interventions, populations, care-journey stages, and outcome domains—information fundamental to responsible research, clinical adoption, and policy development.

### Objectives

Considering this evolving context, we conducted, to our knowledge, the first scoping review conducted in accordance with PRISMA-ScR (Preferred Reporting Items for Systematic Reviews and Meta-Analyses extension for Scoping Reviews) [10] to describe and map the published and gray literature from the past decade on DHTs relevant to LSD care, with a primary analytic focus on AI-enabled and CC applications and a contextual mapping of other enabling DHTs.

**Figure 1:**
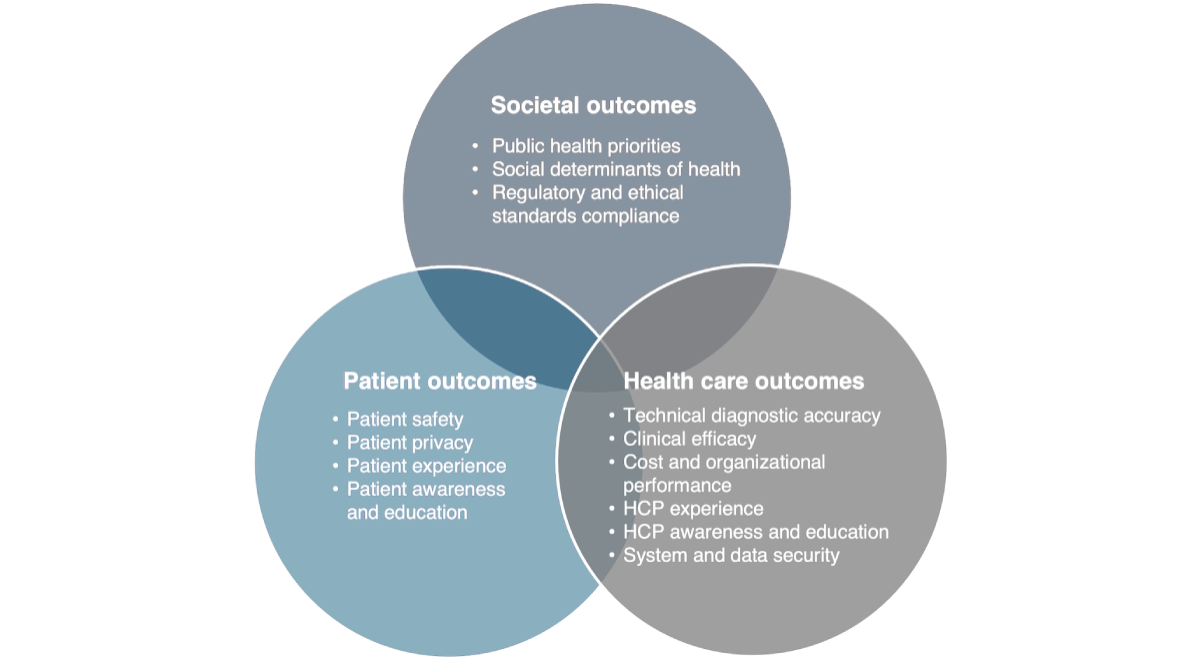
Outcome-based framework to map, cluster, and assess literature for patient, health care, and societal impact. HCP: health care provider.

**Figure 2:**
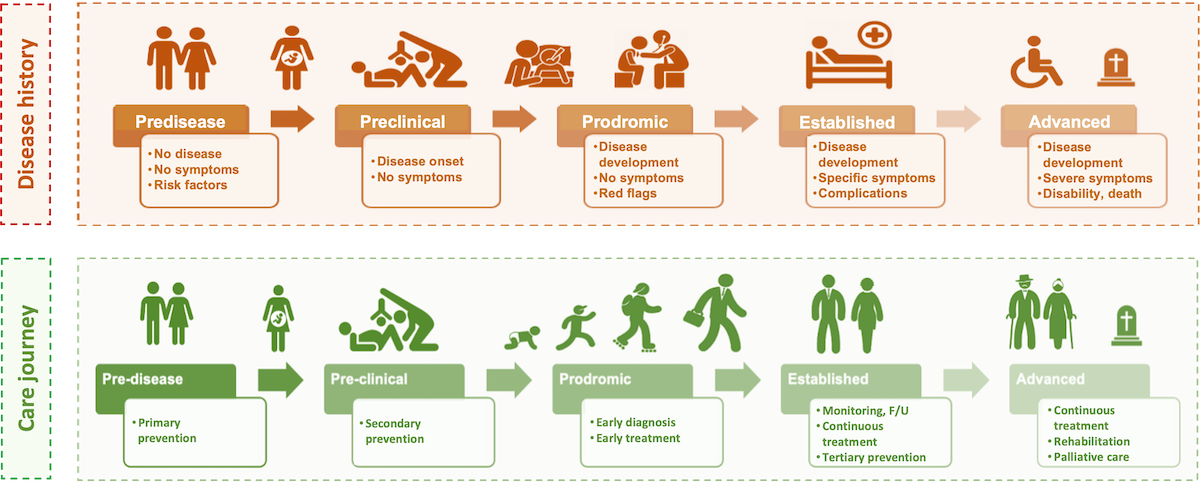
Timed framework to map, cluster, and assess literature for LSD natural history and care journey phases.

Our aims were to (1) clarify scope and applications across AI, CC, and other enabling DHTs; (2) chart the distribution of evidence across 4 axes (populations, intervention types, care-journey phases, and outcome domains) using an explicit charting framework (Textbox 1 and Figures 1-2); and (3) identify gaps, methodological limitations, and research priorities relevant to clinical implementation and policy development [3,5,9].

This review is explicitly descriptive in nature and does not seek to determine effectiveness or infer causal impact.

**Intervention-based categorization framework to map, cluster, and assess literature for AI-driven, connected care, and other digital health technologies.**

**Artificial intelligence–driven digital health technologies**

- Artificial intelligence (AI) algorithms
  Machine learning (ML) models, deep learning (DL) and neural networks (NN), natural language processing (NLP), and large language models (LLMs)
- AI-driven digital health technology (DHT) clinical applications
  Risk stratification, facial recognition, clinical imaging analysis, predictive analytics, decision support, intelligent genomic counselling, virtual agents, and assistants
- AI-driven DHTs research and development applications
  Drug development, enhanced or simulated clinical trials, disease and phenotypic pattern recognition, and disease epidemiological and progression modeling

**Connected care DHTs**

- Telemedicine
  Health care provider (HCP)-patient televisits (including tele-genetics), HCP-HCP teleconsultations, collaborative health care networks
- Digital medical devices
  Remote patient monitoring (RPM) devices, electronic patient-reported outcomes (ePRO), Mobile Health (mHealth) apps, patient care support programs, digital therapeutics (DTx)
- Data integration systems
  Electronic health records (EHRs), health information systems (HIS), and national and international registries
- Patient engagement platforms
  Online patient portals, networks, and peer-to-peer communities

**Other DHTs**

- Clinical DHTs
  Precision medicine platforms, digital microfluidics (DMF) platforms, high-performance liquid chromatography (HPLC)–tandem mass spectrometry (MS/MS), bioinformatics tools, enzyme replacement therapy (ERT) optimization tools, virtual reality (VR) & augmented reality (AR), biomedical imaging, decision support systems (DSS)
- Data integration systems enablers
  Blockchain for health data security
- Research and development DHTs
  Clinical trial simulation tools, computational modeling
  Multipurpose omics and molecular analysis DHTs
  Genomics (including next-generation sequencing), pharmacogenomics, metabolomics, and proteomics applications and platforms.

## Methods

### Overview

The dynamic and rapidly advancing field of DHTs and the variability in the quality of existing evidence render traditional systematic reviews and graded recommendations unsuitable, while making scoping reviews particularly applicable and informative. We used a multidimensional, PICO (population, intervention, comparison, and outcome)-informed, charting framework to classify and map evidence (Textbox 1 and Figures 1-2), while framing the overarching review question using a population–concept–context (PCC) logic. In this study, “PICO-informed” refers to a structured approach to categorize populations, intervention, and outcome domains for mapping purposes, not to a causal comparison framework—consistently with the aim of scoping reviews to map the breadth, characteristics, and gaps of evidence rather than to estimate comparative effectiveness.

The work adheres to the 5-stage scoping review framework advanced by Arksey and O’Malley [11] and follows the PRISMA-ScR guidelines [10,12]. Specifically, reporting is aligned with the 22-item PRISMA-ScR checklist (Multimedia Appendix 1). A prospective protocol was not registered; we acknowledge this as a limitation for transparency and therefore provide full search strings, dates, and flows to support reproducibility.

### Identifying the Research Question

Our review question was as follows: In adult and pediatric populations with LSDs (including at-risk/suspected cases), their caregivers, and/or health care professionals (Population), what types of DHTs, particularly AI-enabled and connected care solutions, along with other enabling digital technologies (Concept), have been reported over the last decade, and how is the evidence distributed across the LSD care-journey phases and across patient, health care delivery, and societal impact domains (Context/Outcomes)?

Comparators were not required for inclusion; when present, they were recorded descriptively. Outcomes were extracted as reported and mapped across predefined domains (Figure 1) to support descriptive synthesis and identification of evidence gaps, rather than causal inference.

### Identifying Relevant Studies

We searched PubMed (including MEDLINE) and Google Scholar as core sources, complemented by queries on ClinicalTrials.gov and AI-assisted discovery tools, to broaden recall beyond records explicitly indexed in the core databases. Filters were applied to include studies published in the last decade, regardless of language, and to capture relevant gray literature, including non-peer-reviewed preprint studies, trial registries, theses, reports, expert interviews, book chapters, websites, and institutional or patient association portals. Overall, the multisource search retrieved 1751 records after prescreening. Full database-by-database reproducibility logs (strings, run-dates, filters, and stepwise counts) are provided in Tables S1-S7 in Multimedia Appendix 2.

On September 30, 2024, the baseline PubMed query returned 218 records (full stepwise string and fixed publication date filter reported in #8 in Multimedia Appendix 2). To increase sensitivity while preserving specificity for LSDs, we expanded the DHT concept using 55 keywords, consolidated from >80 DHT intervention terms identified during preliminary mapping, yielding an additional 190 records (total PubMed yield 408; see #5 and #10 in Multimedia Appendix 2). The term “rare disease” was not included, as it captured an excessive number of papers (>1900) with poor specificity for LSDs.

Google Scholar was searched on the same date using the predefined query and year filter (2015-2024), yielding approximately 4070 results (including duplicates and gray literature). To run specific reproducible screening under platform ranking variability, we applied a prespecified stopping rule by exporting and screening only the first 1000 results sorted by relevance (see #24 and #25 in Multimedia Appendix 2).

A similar research query was run on the randomized controlled trial (RCT) registry ClinicalTrials.gov (full string and fixed publication-date filter reported in #32 in Multimedia Appendix 2). We preliminarily identified 14 ongoing or completed, but unpublished, RCTs.

Consensus, SciSpace, and Connected Papers were used as supplementary AI-driven search tools (query expansion and citation chasing) on September 30, 2024, retrieving respectively 50, 163, and 102 records, including duplicates across the outputs (see #34, #35, and #47 in Multimedia Appendix 2). These tools were used only to identify candidate records; they did not autonomously determine eligibility, extract data, or draw conclusions. For reproducibility, we logged for each tool the run date and reference period, inputs (keywords, filters, or seed articles), and the number of candidate records retrieved. The author team reviewed all retrieved outputs and underwent the same deduplication and screening workflow as all other records.

An additional 14 records were finally added from citations, authors’ suggestions, and general web searches.

### Study Selection

Records were de-duplicated using Paperpile (non**-**AI**,** reference manager software) and then double-checked manually. Title and abstract screening was performed by one reviewer (LN) after calibration with a second reviewer (AMCS) to refine eligibility criteria and resolve ambiguities. To mitigate single-screener selection error, AMCS independently screened (1) all records marked as uncertain and (2) a random sample of excluded records; discrepancies were resolved by discussion and, when needed, adjudication by a third reviewer (MS). LN and AMCS assessed full-text eligibility independently, with MS adjudicating disagreements. All researchers reached consensus on the final selection of studies included for data synthesis.

Given the emerging nature of this research field, the eligibility criteria were kept broad to include all study types published over the past 10 years in any language. Gray literature was also taken into consideration if particularly relevant. However, only records that directly addressed our scoping review question, explicitly involving both LSDs and at least one DHT mapped in our taxonomy (Textbox 1), were selected for analysis.

Inclusion criteria were (1) populations with a confirmed LSD diagnosis or at-risk LSD status (including screening, suspected, or undifferentiated LSD cases), and/or their caregivers or health care professionals; (2) digital interventions classified as AI, CC, or other DHTs according to the taxonomy in Textbox 1; and (3) outcomes that could be mapped to at least one of the predefined patient, health care delivery, or societal impact domains.

We excluded records for which (1) full text, or a minimum set of extractable data, could not be retrieved; or records that were (2) not LSD-specific and/or did not involve a DHT as defined in Textbox 1; where cited in Discussion, it served contextual purposes only. Non–peer-reviewed items were retained only when a stable, traceable source was accessible, and core data could be verified.

### Data Charting and Synthesis

To convey source heterogeneity, we labeled each included record by peer-reviewed and gray source type, and summarized counts and distribution by evidence tier (Table 1). During synthesis, peer-reviewed items were always summarized separately from gray literature. Source validation and interpretive weighting followed a simple hierarchy: peer-reviewed original studies and reviews formed the primary basis for interpretation; gray sources were used descriptively only to contextualize emerging initiatives and evidence gaps. No clinical or system-level statement in this review rests solely on non–peer-reviewed sources.

**Table 1:** Literature source types of included records (distribution): corpus split into peer-reviewed and gray literature with subcategories per tier.

Codes and source type				Unique papers, n		% Source tier^a^, % (n/N)		% Total papers^b^, % (n/N)		Role in synthesis
**L.1 Peer-reviewed**				226		100 (226/226)		92.2 (226/245)		—^c^
	L.1.1	RCTs^d^, systematic reviews (SR), meta-analyses	0		0		0		Primary	
	L.1.2	Narrative, scoping reviews	44		19.5 (44/226)		18 (44/245)		Secondary	
	L.1.3	Original research (non-RCT)	176		77.9 (176/226)		71.8 (176/245)		Secondary	
	L.1.4	Case reports, case series	6		2.7 (6/226)		2.4 (6/245)		Context only	
	L.1.5	Editorials, letters, commentaries, perspectives	0		0		0		Context only	
**L.2 Gray literature**				19		100 (19/19)		7.8 (19/245)		—
	L.2.1	Registered, ongoing RCTs	7		36.8 (7/19)		2.9 (7/245)		Context only	
	L.2.2	Preprints	2		10.5 (2/19)		0.8 (2/245)		Context only	
	L.2.3	Conference abstracts, proceedings	1		5.3 (1/19)		0.4 (1/245)		Context only	
	L.2.4	Theses, dissertations	4		21.1 (4/19)		1.6 (4/245)		Context only	
	L.2.5	Books, book chapters	2		10.5 (2/19)		0.8 (2/245)		Context only	
	L.2.6	Websites, web posts	1		5.3 (1/19)		0.4 (1/245)		Context only	
	L.2.7	Reports, white papers, interviews	1		5.3 (1/19)		0.4 (1/245)		Context only	
	L.2.8	Patents	1		5.3 (7/19)		0.4 (1/245)		Context only	
L.1+L.2 All included records				245		—		100 (245/245)		—

^a^% of Total Papers uses 245 as the denominator.

^b^% of Source Tier uses 226 for peer-reviewed (182 research articles + 44 reviews) and 19 for gray literature (7 registered RCTs + 12 miscellaneous sources).

^c^Not available.

^d^RCT: randomized controlled trial.

In line with scoping review guidance, and due to the heterogeneity of study designs, objectives, and sources of evidence, no formal assessment of the risk of bias or methodological quality was performed. Instead, we charted design, sample size, setting, and other quality-relevant features and used these descriptively to temper interpretation in the Results and Discussion sections.

Evidence was mapped and synthesized along the 4 dimensions derived from our multidimensional review question: Population, Intervention class (Textbox 1), Outcome domains (patient, health care delivery, and societal-level outcomes; Figure 1); and Care**-**journey phase (Figure 2). This population–intervention–outcome–care-phase analytic framework accommodated heterogeneous designs; it was descriptive and label-based, relying on counts and cross-tabulation rather than formal meta-analysis or effect-size estimation.

Population in scope was represented by adults and children diagnosed (or at-risk of being affected) with LSDs, with a particular focus on Gaucher disease (GD), Fabry disease (FD), mucopolysaccharidosis (I, II, IIIA, IIIB, IVA, VI, VII), Pompe disease (PD), Niemann-Pick disease, Krabbe disease, metachromatic leukodystrophy, GM1 and GM2 Gangliosidosis (GM1G and GM2G), Batten disease, and aspartylglucosaminuria, among more than 70 classified entities. Where possible, insights were charted and reported by age group and LSD subtype to reflect differences in clinical presentation and care needs.

We clustered DHT interventions into 3 domains that were applied consistently during study selection, charting, and synthesis: AI-driven DHTs, CC DHTs, and other DHTs (Textbox 1). AI-driven DHTs are adaptive and, to varying degrees, autonomous systems capable of learning, reasoning, and generating outputs from input data (eg, machine learning [ML] and deep learning [DL] models, natural language processing [NLP and large language models [LLMs]). In clinical contexts, these tools typically support early diagnosis and phenotyping, risk prediction, and decision support. CC DHTs include telemedicine; DMDs such as devices monitoring clinical parameters and electronic patient-reported outcomes (ePROs), mobile health (mHealth) apps, and digital therapeutics (DTx); interoperable data-integration systems (electronic health record [EHR], health information systems [HIS], registries, and emerging health-data spaces); and patient-engagement platforms. The category of other DHTs comprises clinical and research and development (R&D) tools that are neither AI-based nor connected-care systems (eg, digital microfluidics, omics and bioinformatics platforms, non-AI imaging tools, and trial-simulation software).

The impact of DHTs was then assessed across outcomes grouped into 3 domains (Figure 1): patient-related outcomes (patient safety, privacy, experience, awareness and education), health care delivery outcomes (technical diagnostic accuracy, clinical efficacy, cost and organizational performance, system and data security, health care provider [HCP] experience, awareness and education), and societal outcomes (public health and social determinants of health, regulatory and ethical compliance). Each included record was coded to one or more (≥1) outcome domains; when a study reported multiple domains, it was multilabeled. As a result, totals in outcome tables may exceed the number of unique records. The Discussion interpreted cross-domain patterns (rather than effectiveness) in line with scoping aims.

We also generalized the natural history of LSDs and summarized how DHTs were positioned across key care-journey phases**:** primary prevention, secondary prevention (screening) and diagnosis, treatment, tertiary prevention and rehabilitation, monitoring and follow-up, and end-of-life care (Figure 2; Multimedia Appendix 3). Each included record was mapped to ≥1 phase depending on where in the journey the intervention (AI, CC, or other DHTs) was evaluated or deployed; multiphase studies were multicoded. The visual representation does not imply causation; it provides placement along the pathway to support descriptive synthesis in the Results and higher-order interpretation in the Discussion.

To interpret plausible causal pathways and specify testable hypotheses for LSD care, we also drew on evidence from adjacent non-LSD clinical domains where AI (eg, diagnostic triage and risk prediction) and CC (eg, televisit, teleconsultation, remote follow-up and monitoring adoption, and adherence) have been more extensively studied. These analogies were cited only as contextual signals (not pooled with LSD-specific data) and used to motivate prospective evaluations (eg, multisite designs, external validation, and standardized outcomes) in LSD settings.

Along the charting process, risks, challenges, and biases associated with DHTs, evidence limitations, and future research needs were identified and reported in the Results and Discussion sections. Key examples are also summarized in Multimedia Appendix 4.

## Results

### Overview

**Figure 3:**
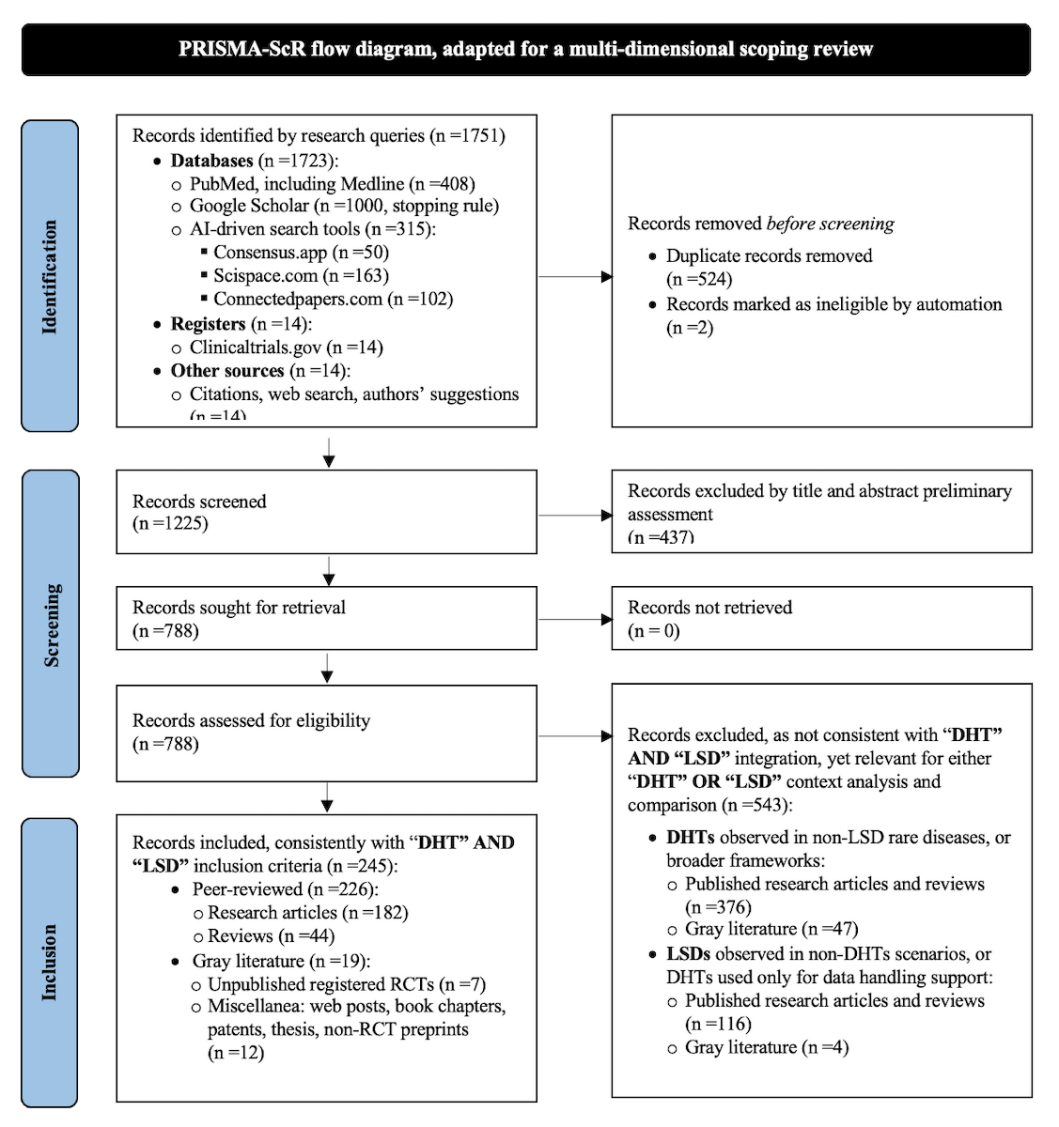
PRISMA-ScR (Preferred Reporting Items for Systematic Reviews and Meta-Analyses extension for Scoping Reviews) flow diagram adapted for a multidimensional scoping review and evidence-mapping framework [9]. AI: artificial intelligence; DHT: digital health technology; LSD: lysosomal storage disorder; RCT: randomized controlled trial.

After the identification of 1751 records, we applied a rigorous selection process adapted to the nature of this multidimensional scoping review and mapping framework (PRISMA-ScR flow in Figure 3). The screening first led to 1225 items after removing duplicates and ineligible entries. The following screening process on titles and abstracts led to the exclusion of 437 records that did not meet relevance criteria coherent with the review question and eligibility criteria. A detailed assessment was then conducted on 788 records. Thorough eligibility assessment ensured each document was relevant to the specific focus, by embracing both LSDs management and DHTs integration, resulting in 245 records being included for final consideration: 182 research articles, 44 reviews, 19 “grey” literature records (including 7 ongoing or unpublished registered RCTs and other sources such as preprints, thesis, book chapters, periodicals, reports, and web posts). Finally, within the peer-reviewed literature, 40 items focused on AI-driven DHTs, 89 on CC DHTs, and 144 on other enabling DHTs, with some reporting more than one category. Nearly all records were published in English, only a few in Italian, German, and Russian.

In addition to LSD-focused records, the searches surfaced many articles on digital health applications in other rare and non-rare conditions. A subset of 492 journal articles and 51 pieces of gray literature was consulted only for contextual illustration. However, these contextual references were not systematically reviewed and did not influence study selection, data extraction, or synthesis.

Data were summarized in a PRISMA-ScR flow diagram (Figure 3), reporting identification, screening, eligibility, and inclusion counts for the corpus. Numbers correspond to records after deduplication and reflect all intervention classes (AI, CC, other DHTs). Reasons for exclusion at full text are summarized in the right-hand box and detailed in the Methods section. Transparency is provided via full search strings, logs, and the PRISMA-ScR checklist (Multimedia Appendix 1).

Expanded study-level evidence charting tables, showing distribution across literature sources, and comparing population characteristics, DHT categories, and care-journey mapping, are provided in Table 1 and Multimedia Appendix 5.

A synthesis at-a-glance of literature source distribution (Table 1) shows that, across the 245 included records, more than 90% (226/245) are peer-reviewed journal items and 7.8% (19/245) are gray literature (registered or ongoing trials, theses, reports, web-based documents, and other non–peer-reviewed sources). No completed RCTs, systematic reviews, or meta-analyses with LSDs-specific digital interventions were identified. Instead, the corpus is dominated by original observational, nonrandomized research and narrative or scoping reviews, with a small tail of case reports and contextual gray sources. Consistent with our scoping aim, peer-reviewed studies form the core basis for interpretation, whereas gray sources are used descriptively to map context and evidence gaps.

Disease-level comparative labeling (Table S1 in Multimedia Appendix 5) shows that evidence is unevenly distributed across LSDs. GD and FD together account for nearly 60% (130/226) of peer-reviewed papers, with additional clusters in mucopolysaccharidosis and PD, and only sparse coverage for other LSDs. To avoid overgeneralization, our synthesis is weighted by this coverage: where an LSD has multiple, convergent peer-reviewed studies, we treat findings as relatively more robust; where counts are very low, we label insights as signal-only and refrain from extrapolating cross-disease claims. This approach preserves disease-specific detail while explicitly acknowledging both sounder evidence and gaps.

Standardized intervention labeling (Table S2 in Multimedia Appendix 5) yielded 380 labels across AI, CC, and other DHTs. Although AI-specific labels represent only about 14% (52/380) of all intervention labels, they appear in approximately 18% (40/226) of peer-reviewed papers, largely in diagnostic and clinical-decision support applications. CC labels account for 32% of all labels (122/380), with DMDs and telemedicine applications most frequently represented. The remaining half of labels fall under other, mostly enabling DHTs (eg, omics platforms, bioinformatics pipelines, and non-AI imaging tools), which provide critical infrastructure. Since multicategory records were multicoded, labeled-paper totals may exceed; we therefore interpret these distributions qualitatively as relative signal strength rather than prevalence estimates.

Outcome-domain labeling (Table S3 in Multimedia Appendix 5) yielded 430 outcome labels across 245 records. Roughly two-thirds of labels (279/430, 64.9%) fall within health care delivery outcomes, led by technical diagnostic accuracy (157/430, 36.5%), with cost and organizational performance plus system and data security together contributing a further 75/430 (17.4%) labels. Patient-level outcomes account for just over one quarter of labels (117/430, 27.2%), dominated by patient experience (45/430, 10.5%) and privacy (39/430, 9.1%), whereas patient safety, awareness, and education remain relatively infrequent (33/430, 7.7% combined). Societal outcomes are least represented, with only 34 of 430 labels (7.9%), 10 of 430 (2.3%) for public health and social determinants of health, and 24 of 430 (5.6%) for regulatory and ethical compliance. Technical diagnostic accuracy alone accounts for more than one-third of all outcome labels and about half of peer-reviewed outcome reports, underscoring a strong focus on accuracy, detection, and classification—for now, more than on clinical efficacy and long-term patient or societal impact. This distribution reflects an evidence landscape characteristic of emerging innovations, in which establishing technical reliability and safety benchmarks precedes the generation of robust data on clinical effectiveness, patient-centered outcomes, and broader societal impact.

Care-journey mapping (Table S4 in Multimedia Appendix 5) shows that nearly half of all phase labels (222/472, 47%) concentrate in secondary prevention (screening) and diagnosis, followed by monitoring and follow-up (116/472, 24.6%) and treatment (95/472, 20.1%), while preconception primary prevention accounts for only 11/472 (2.3%) labels. Rehabilitation, tertiary prevention, and end-of-life care together contribute 28/472 (5.9%) labels, indicating much slimmer digital activity in later or more complex phases of the care journey. Across phases, other DHTs dominate diagnostic and treatment phases (eg, 120/222 labels, 54.1%, in secondary prevention and diagnosis), whereas CC labels are relatively more prominent in monitoring and follow-up (47/116, 40.5%), and AI labels remain numerically modest in downstream phases. These patterns, aligned with the timed framework in Figure 2, highlight a key digital footprint and exploration of applications to enhance early diagnosis and ongoing monitoring, with comparatively fewer digital interventions evaluated in primary prevention and in later phases of the care journey. The subsequent Results subsections unpack these phase-specific signals descriptively, while the Discussion interprets their implications for evaluation and implementation.

### Population-Specific Insights by Disease

#### Coverage Patterns Across LSDs

As shown in Table S1 in Multimedia Appendix 5, disease coverage is highly asymmetric: FD and GD account for >50% of peer-reviewed records, whereas most other LSDs have limited or no DHT literature. We therefore synthesize findings by LSD and avoid cross-disease inference when evidence is sparse.

Although age was not charted as a separate axis, several records describe distinct digital use-cases in pediatric vs adult cohorts. In pediatric care, tools emphasize early detection (eg, neonatal blood spot screening and facial recognition), adherence and progression monitoring, and child- and family-centered education (including virtual reality [VR] and gamified approaches), alongside concerns about limited evidence, access inequities, and privacy [13-20]. In adults, mHealth apps, portals, and telemedicine emphasize self-management and access, while analytics support personalized planning and detection of late-onset manifestations [21-23]. Data scarcity, heterogeneity, and privacy governance recur across age groups [13-23].

#### Fabry Disease

In FD, AI use-cases cluster around early detection, phenotyping and organ involvement: facial recognition and image analytics (eg, magnetic resonance imaging [MRI], urinary sediment imaging, electrocardiography, echo, and MRI) to detect brain, renal, and cardiac manifestations, and support prognosis, plus EHR-based ML to flag high-risk patients for screening [24-33]. Variant-level prediction of pharmacologic responsiveness from structural and proteomic features is also reported [34-37].

CC and enabling tools support longitudinal management through telemedicine, DMDs, ePROs, registries, and EHRs for symptom tracking, adherence support, and remote follow-up, including app-based support programs integrating wearable or patient-entered data [22,38-43]. Enabling diagnostics include non-AI EHR risk models, digital microfluidics (DMF) + tandem mass spectrometry (MS/MS) newborn screening, and next-generation sequencing (NGS)–based confirmation and precision workflows [14,44-47]. Reported risks include overreliance on digital outputs and discordance with patient experience and clinical status [38,48,49].

#### Gaucher Disease

In GD, AI is most often applied to imaging-based phenotyping (eg, MRI-derived bone complication prediction; automated spleen-volume estimation) and to EHR and registry analytics supporting risk stratification and screening [8,22,44,50-59]. CC components include televisits, teleconsultations (highlighted during COVID-19) and, in some records, wearables, mHealth, and ePROs for intervisit monitoring of gait, sleep, pain, and stress [6,7,22,60,61]. Enabling diagnostics include genomic-analysis workflows and DMF-based assays supporting mutation detection and newborn-screening pathways [62-64]. Reported limitations include small datasets, bias risk, and limited evidence on long-term impact [56].

#### Mucopolysaccharidosis

For mucopolysaccharidosis, records describe ML-supported differential diagnosis and prediction of treatment response, complemented by CC (telemedicine and tele-genetics) for remote consultation, adherence monitoring, and education [22,60,65-67]. Enabling tools include genomic pipelines for mutation detection and DMF platforms for diagnostic assays [47,68]. Reported challenges include data integration, regulatory compliance, and evolving practices that require ongoing updating of digital tools [67,69].

#### Pompe Disease

For PD, AI-enabled tools are reported for EHR-based case finding and progression prediction, and for variant, enzymatic analytics within multiomics research pipelines [35,70-73]. CC applications include telemedicine-supported physiotherapy supervision and cognitive training, plus mHealth trackers and ePROs to monitor neuromuscular function and correlate patient-reported metrics with clinical outcomes, particularly in late-onset PD [60,74-79]. Genomic workflows support mutation identification and personalization [47,80]. Reported implementation barriers include technical and regulatory constraints, costs, fragmented care pathways, and variable user acceptance [74,75,81].

#### Other LSDs

Beyond FD, GD, mucopolysaccharidosis, and PD, evidence is sparse and typically disease-specific.

In Niemann-Pick disease, AI-driven tools and metabolomic profiling enhance diagnostic accuracy, and DMF platforms streamline diagnostic assays [68,82,83].

Computational tools enhance newborn screening in Krabbe disease by improving specificity and reducing costs, effectively differentiating true-positive from false-positive cases [84].

In MLD, AI algorithms aid in quantifying demyelination load in MRI scans [85,86] and simulate disease mechanisms to predict drug efficacy before clinical trials [87].

Telehealth services improve health care access and satisfaction for patients with Batten disease, enhancing care delivery and potential long-term benefits [20].

MRI scoring and quantitative spectroscopy can be used as surrogates of clinical disease progression in GM1G, as they correlate with ambulation and expressive language gradual impairment [88]. Wearable devices and ePROs may monitor patients with GM2G remotely, assessing health aspects like fatigue, stress, and self-esteem [89].

ML analysis of thalamus MRI images may diagnose aspartylglucosaminuria in patients with neurocognitive symptoms like progressive learning disabilities, with low error rates and promising diagnostic potential [90].

### Intervention-Specific Insights by AI-Driven DHTs

#### Overview

As summarized in Table S2 in Multimedia Appendix 5, intervention subtypes by record, AI-driven DHTs constitute one of the 3 main classes of interventions mapped in this review, with studies clustering predominantly around diagnostic decision support, image and signal analysis, risk prediction, and data-driven patient stratification. The subsections below provide illustrative examples of how AI is being applied along LSD care pathways, highlighting typical use-cases rather than attempting a formal performance ranking.

#### AI for Risk Prediction and Patient Stratification

ML models identify patients at risk of developing specific diseases, predict the risk of complications, and stratify patients based on disease severity. This is generally achieved by analyzing patient demographics, clinical features, and genetic information [30,56,59,71].

#### AI for Clinical Diagnostics and Therapeutics

ML models identify potential LSD drug candidates by screening compound libraries and matching data against protein or genomic patient-specific data. They optimize treatment strategies, such as enzyme replacement therapy (ERT), and predict the responsiveness of individual variants to specific treatments [8,28,35,37,52,56]. AI-powered DSS shows use in metabolic conditions where overlapping symptoms often cause diagnostic delays. Intelligent systems may achieve high accuracy (87.4%) in differential diagnosis of LSDs by generating a list of diagnostic hypotheses based on clinical data [16,91,92].

DL algorithms, as a subset of ML that uses artificial neural networks with multiple layers to analyze complex patterns, are widely used for analyzing medical images (eg, brain or cardiac MRI and computed tomography, echocardiography, electrocardiogram, tissue or blood smears, facial features) to detect clinical biomarkers, structural anomalies, and disease-specific LSD patterns. For example, AI applications improve early detection of spleen and liver enlargement [54] or osteonecrosis in GD [51-53], or brain [26,90] and cardiac [28-32,34,93-95] involvement, mostly in FD and PD. AI-driven facial recognition software, used in diagnosing GD and FD, detects distinctive facial features commonly presented in large patient cohorts with early diagnosis potential [24,96,97].

NLP and LLMs extract critical information from unstructured data sources, such as clinical notes, interviews, research papers, insurance claims, and other textual sources. These insights can be integrated into AI-powered symptom assessment or DSS, informing diagnostic and treatment support for LSDs, particularly where clinical expertise is limited or symptoms are challenging to diagnose. NLP enables computers to understand and process human language, allowing them to extract meaningful insights from textual data, identifying patterns and correlations. LLMs have demonstrated remarkable capabilities in understanding and generating human-like text, summarizing patient narratives, extracting key medical concepts from clinical notes or visits, and generating comprehensive reports [33,98,99].

#### AI for R&D

There are already significant applications of ML and DL models in research on LSDs, including disease modeling, clinical trial simulations, drug discovery, and data analysis. ML tools identify disease-relevant compounds, predict their potential as inhibitors or chaperones, and link them to disease targets, advancing R&D efforts [37,72,73,100,101]. DL and facial recognition software aid in the investigation and analysis of facial phenotypes in patients with LSDs, contributing to the understanding of these RDs [21,102]. Researchers use AI and simulations to study lysosomal enzymes and their role in storage diseases. These tools may speed up drug development, especially for gene therapies and targeted treatments. AI may optimize preclinical trials and simulate disease progression and treatment response, predict how diseases may evolve, explore new treatments, and simulate genetic mutations and their effects, aiding in establishing standardization and personalization workflows.

### Intervention-Specific Insights by CC DHTs

#### Overview

As summarized in Table S2 in Multimedia Appendix 5, CC solutions are most often used to extend specialist input across distance, support long-term follow-up, and coordinate multidisciplinary care around complex, chronic trajectories. The narrative examples below illustrate how teleconsultation, home-based and hybrid models of care, and registry- or platform-based coordination are being deployed in practice, while acknowledging that formal comparative, cost-effectiveness, and equity analyses remain limited.

#### Telemedicine

Televisits among providers and patients (HCP-patient) enhance LSD care delivery. Evidence highlights the key role televisits played during the COVID-19 pandemic when in-person consultations were limited [6,15,17,39-41,60,74,103-112].

Platforms supporting virtual visits, genetic counseling (tele-genetics), and real-time monitoring have proven invaluable for maintaining continuity of care, particularly for patients in remote or underserved areas. For instance, telemedicine ensured that patients receiving ERT for conditions like GD and FD continued to receive necessary follow-ups, reducing “missed appointments” rates. Tele-genetics has specifically improved access to genetic testing and counseling, enhancing screening, early diagnosis, and treatment planning [20,43,66,67,75,113].

Tele-consultations among HCPs and collaborative health care networks enable multidisciplinary teams, including geneticists, pediatricians, cardiologists, neurologists, nephrologists, and primary care providers, to discuss complex cases. This collaborative approach has been crucial for LSDs like GD and FD, where care often requires input from various specialists, and nonspecialized professionals may need support from expert centers to enhance early diagnosis or continuous care. European Reference Networks already foster multidisciplinary care for RDs and, specifically, for LSDs through cross-border collaboration and could be enhanced by telemedicine and digital health applications [105,114-116].

#### Digital Medical Devices (DMDs)

Certified medical device software, such as remote patient monitoring devices, including medical-grade wearables and mHealth apps supported by proof of clinical efficacy, allows for reiterative tracking of real-world inter-visit biomarkers, like heart rate variability, respiratory function, mobility (including gait and fatigue patterns), providing early warnings for disease progression and symptom exacerbations. Examples include monitoring cardiovascular health for FD and activity trackers to help measure physical function as a surrogate for disease progression in GD, FD, PD, and G2MG [7,43,61,76-78,89].

Monitoring of ePROs has also demonstrated efficacy in collecting long-term data from patients with RDs, providing HCPs with detailed insights into daily symptom variations. For instance, patients with FD use ePROs to report neuropathic pain symptoms, enabling clinicians to timely adjust pain management protocols [43,49,61,78,79,89,117-121].

The adoption of digital patient care support programs, often delivered through mHealth applications, correlates with better adherence to management and treatment plans. They empower patients to take an active role in managing their care, leading to improved outcomes. Studies have shown that mHealth apps enhance patient experience, medication adherence, and help track daily symptoms, which are crucial for the long-term care of patients with LSD [7,41,43,61,76-78].

Safe and effective DTx [122] may enhance care through digitally-delivering treatments for common LSD symptoms and comorbidities, in particular, psychological symptoms, such as depression and sleeping disorders [123]. Digitally delivered cognitive behavioral-based interventions are already recommended in recent depression and insomnia treatment guidelines [124,125] and in Germany, as of December 2025, 29 DTx for mental health are already reimbursed and can be prescribed, 3 of which for sleeping disorders [126].

#### Patient Engagement Tools

Patient portals and online patient networks and communities have fostered greater patient engagement, particularly for RDs like LSDs. In managing long-term conditions like FD, patients who regularly access portals to review their health records and appointments are more likely to adhere to their treatments [8,57,117]. While it is important to ensure correct and up-to-date information, social platforms have created spaces for patients with LSDs to share experiences, discuss treatment side effects, and find emotional support, which in turn improves overall adherence to therapy.

#### Health Care Data Integration Systems and Disease Registries

Shared EHRs, HIS, and patient registries enhance RDs’ care and research, and enhance multidisciplinary collaboration across care teams and disease networks. In LSD management, EHRs allow geneticists, nephrologists, and other specialists to collaborate on treatment plans, streamlining the care process and improving decision-making. Research highlights that EHRs reduce the likelihood of miscommunication, which is critical for patients requiring long-term monitoring and multiple specialists [44,55,58,70,71].

Integrated international registries, often reported among the sources of published studies, have contributed significantly to understanding disease progression and treatment response in LSDs. These interfaces facilitated data sharing across health care institutions and HCPs, improving research into RDs by offering large-scale epidemiological data on disease prevalence and long-term outcomes [2,22,38,59,118,127-134].

### Intervention-Specific Insights by Other DHTs

Beyond AI and CC, other enabling DHTs underpin much of LSD diagnostics and research and often supply the data and infrastructure that later feed AI or CC applications (Table S2 in Multimedia Appendix 5). Key examples include: high-throughput biochemical diagnostics (DMF and advanced MS/high-performance liquid chromatography-MS/MS workflows), genomics and pharmacogenomics pipelines, bioinformatics and multiomics platforms, digitized imaging and 3D modeling, VR and augmented reality education, selected cognitive and rehabilitation use-cases, non-AI DSS, secure data architectures (including blockchain) for registry- and trial-enabling data sharing, and computational and trial-simulation models for progression and study-design optimization [10,13,16,42,46,47,62,64,68,72,73,80,83-87,92, 100,101,135-156].

### Outcome-Specific Insights by Domain

#### Overview

Table S3 in Multimedia Appendix 5 summarizes how outcomes were labeled across the 245 records, grouped into patient, health care delivery, and societal domains. Across all intervention classes, 65% of labels fall within health care delivery outcomes, 27% within patient outcomes, and fewer than 10% within societal outcomes. The following subsections provide a detailed, outcome-based synthesis of the results, grouped across the 3 predefined key domain areas.

#### Patient Outcome Domain

At the patient level, labeled outcomes mainly address safety, privacy, experience awareness, and education, and are less frequently represented than health care delivery outcomes. AI-enabled predictive analytics, telemedicine, and remote-monitoring DMDs are typically assessed for their ability to anticipate deterioration, flag early warning signs, and reduce avoidable unscheduled visits or admissions in chronic LSD manifestations (eg, cardiomyopathy and skeletal complications) [41,55,61]. Patient experience is usually captured through satisfaction, convenience, perceived access to expertise, usability of telehealth and mHealth tools, while privacy, awareness, and education outcomes appear in studies on consent, information provision, and trust in digital platforms [43,49,60,61,63,78,79,103,108,118,120,157,158].

Observational studies and pilots report that AI-driven risk-prediction tools, telemonitoring platforms, and wearable DMDs are used to identify early signals of deterioration or treatment-related complications, with outcomes reported as fewer emergency contacts, earlier detection of complications, or improved adherence to safety-monitoring protocols [7,41,55,61]. However, safety signals are predominantly process-oriented (alerts generated; actions triggered) rather than hard clinical endpoints, and sample sizes remain small. Reported risks include alert fatigue, overreliance on algorithm outputs, and missed events due to connectivity or data-quality failures [20,41,61].

Privacy outcomes are mostly described through technical and governance safeguards (encryption, access control, and occasionally blockchain-based architectures for registry and genomic data) and through documentation of GDPR-aligned policies, consent capture, and cross-border data-flow governance [63,103,108,152,153,157]. Direct assessment of privacy perceptions is less common, but some reports note that transparency on data use and clear governance arrangements support sustained trust in AI and CC deployments [21,60,69,159,160].

Telemedicine visits, home-based ERT programs, mHealth apps, wearables, VR tools, and online communities are frequently evaluated via patient experience and satisfaction endpoints, with commonly reported benefits including reduced travel burden to expert centers, perceived continuity of specialist input, and more flexible scheduling and follow-up [43,60,61,76-79,118]. Remote monitoring and digital support programs are also linked to perceived gains in self-management and shared decision-making, while digital literacy, connectivity, and device access can limit uptake and drive preference for hybrid models combining in-person care with digital touchpoints [20,60,69,107].

Digital support programs, disease-specific portals, and AI-enhanced educational tools (eg, symptom-assessment apps and interactive e-clinics) deliver tailored information on disease mechanisms, treatment options, and self-management; outcomes typically include self-reported improvements in knowledge, confidence, and engagement with care plans [20,33,99,107,116,117,158]. Online communities and social media groups may add peer-to-peer support, but raise concerns about variable information quality and the need for expert moderation [69,117].

#### Health Care Delivery Outcome Domain

Health care delivery outcomes account for nearly two-thirds of all outcome labels, dominated by technical diagnostic accuracy, clinical efficacy, system and data security, cost and organizational performance, and health care provider experience and education. AI, CC, and other DHTs are most evaluated on how they improve detection and classification, streamline workflows, support multidisciplinary coordination, and integrate data across settings [46,51-54,68,70,80,97,102,128,134,161].

Security outcomes are reported mainly for registries, EHR-linked algorithms, telemedicine platforms, and blockchain-based prototypes [63,70,103,108,128,152,153]. Evaluations focus on adherence to security standards (encryption, authentication, and audit trails), resilience to cyber-risks, and governance arrangements for access control and data sharing. Some registry and blockchain initiatives explicitly frame security and privacy as prerequisites for cross-border research collaboration and patient trust, but empirical testing of security robustness is rarely detailed beyond high-level descriptions [69,128,152,153].

Several reports and value-based care initiatives describe gains in organizational performance from telemedicine, home-based ERT, digital registries, and automated data flows, including reduced travel and visit burden, fewer missed appointments, and perceived efficiencies in multidisciplinary coordination [20,23,50,76,116,128,134,161]. Cost-related outcomes are reported as descriptive estimates of resource savings (eg, avoided hospital visits and reduced inpatient days) or as qualitative assessments by clinicians and managers. Up-front investment needs, maintenance costs, and training requirements are also highlighted, particularly in under-resourced settings [23,69,159,161].

HCP experience outcomes concern the usability and perceived value of teleconsultation networks, interoperable HER and registry platforms, and DSS (AI-enabled or rule-based) [16,99,116,139,146,149,150]. Reported benefits include faster access to expert opinions, reduced professional isolation, and more structured approaches to complex diagnostic workups. At the same time, several studies note workflow disruption, documentation burden, and uncertainty around medicolegal responsibility as barriers to sustained adoption, especially when digital tools are introduced without sufficient co-design and training [116,149,162,163].

Digital education platforms, simulation tools, and decision-support interfaces are used to disseminate guidelines, RD curricula, and case-based learning for LSDs and other RDs [116,139,146,149,158]. Outcomes commonly include self-reported changes in knowledge, confidence, or diagnostic accuracy in simulated scenarios. Evidence on long-term behavior change or patient-level impact is limited, but these initiatives are frequently cited as enablers for broader DHT uptake and for strengthening expertise in non-specialist settings.

#### Societal Outcome Domain

Societal outcomes are least represented and cluster into public-health and social-determinants impacts, and regulatory and ethical compliance, appearing mainly in policy reports, registry initiatives, and mixed-methods studies on access, equity, and governance [1,60,69,153,160]. Public health endpoints are described primarily for telemedicine, networked registries, and outreach programs (eg, geographic reach, referral patterns, and continuity of care during disruptions), with digital divides in connectivity, device access, and digital literacy as recurrent constraints [1,6,60,69,105,109,130,153]. Regulatory and ethical outcomes focus on alignment with evolving frameworks for AI, medical devices, HTA, and data protection (including EU AI Act and national DTx pathways), emphasizing transparency, consent, privacy, and postmarket oversight while noting limited empirical evaluation of regulatory impact [9,21,38,122,126, 128,152,153,160,162-164].

### Outcome-Specific Insights by Care Journey Phase

#### Overview

Table S4 in Multimedia Appendix 5 and Figure 2, integrated by Multimedia Appendix 3, summarize how AI, CC and other DHTs applications and relative research are distributed along the LSD care journey; the paragraphs below provide a descriptive narrative of how these technologies are deployed across primary prevention, secondary prevention and diagnosis, treatment, monitoring and follow-up, rehabilitation, tertiary prevention, and end-of-life care, without inferring comparative effectiveness.

#### Primary Prevention

Primary prevention evidence is sparse and centers on preconception genetic risk identification. Genomic screening pipelines (including NGS, pharmacogenomics, and related digital platforms) and, in some reports, AI-enabled analytics support risk assessment and counseling, including via tele-genetics [14,45-47,62-64,66,68,80,84,165,166]. Ethical considerations include privacy and genetic discrimination risks.

#### Secondary Prevention and Diagnosis

Across the included studies, AI is used mainly for diagnostic support and phenotyping (eg, facial analysis; imaging analytics across MRI, CT, ultrasound, and smears; EHR-based case finding and severity prediction) [16,24,26,31,33,70,91,92, 97-99,102,167]. A smaller set of papers reports NLP and LLM approaches to extract clinically relevant signals from unstructured text to support diagnostic reasoning [33,98,99]. CC solutions complement this pathway through televisits or teleconsultation and EHR-linked data integration, including ePRO and mHealth streams that support longitudinal assessment and diagnostic refinement, particularly for geographically dispersed families [6,20,40,43,60,61,66,76,89].

In secondary prevention and diagnosis, digital tools cluster around high-throughput screening, confirmatory pipelines, and decision support. DMF and related biochemical screening technologies underpin prenatal and neonatal screening and can accelerate early identification of disorders such as FD and GD [14,45]. Genomic workflows (NGS and other omics pipelines) support mutation detection or confirmation, and precision profiling, supplying structured data streams to downstream analytics [46,47,80,168].

#### Treatment

Treatment-phase applications emphasize personalization and longitudinal adjustment. AI-enabled analytics leverage genomic, proteomic, and metabolomic inputs to support therapy tailoring and to model treatment responsiveness, with reports spanning compound discovery and variant-level prediction [8,28,35,36,72]. CC components (televisits, mHealth, and teleconsultation) support timely initiation and adjustment of therapies such as ERT or substrate reduction, facilitate follow-up and multidisciplinary coordination, and perform continuous-care models integrating ePROs and biometric data for responsive management in conditions such as PD and FD [22,39-41, 60,74,76,169].

#### Monitoring and Follow-Up

For long-term follow-up, CC and monitoring DHTs enhance assessment from episodic visits to inter-visit real-world data. Remote-monitoring DMDs, wearables, ePROs, and digital patient support programs are used to track multidimensional manifestations (eg, cardiac and respiratory function, mobility and gait, sleep, pain, and mood), enabling timely treatment adjustments and adherence support [7,40,43,61,76-78]. Telemedicine and teleconsultation platforms extend specialist oversight and multidisciplinary coordination, while EHR-linked integration (and, in some reports, AI-enabled analytics) consolidates wearable and clinical data into more holistic care plans, particularly for chronic complications such as cardiomyopathy in FD and skeletal disease in GD [22,29,33,51].

#### Rehabilitation

Evidence in rehabilitation is limited, but suggests that mHealth apps, patient support programs, tele-rehabilitation platforms, and selected DTx may support at-home therapy and monitoring, sometimes integrating psychological support [61,76-78,118, 123,148]. The literature emphasizes the need for clinical validation and implementation integration before these approaches can be scaled [69].

#### Tertiary Prevention

Tertiary-prevention records mainly describe remote monitoring tools (wearables and apps), and, in some cases, AI-enabled DSS to adjust care plans using real-world markers to prevent complications [71,76-78,89,128]. Blockchain is discussed to strengthen privacy and trust for long-term data sharing; equity, accessibility, and preservation of human contact are recurrent implementation considerations [38,69,103].

#### End-of-Life Care

End-of-life care evidence is limited, but suggests that telemedicine and remote monitoring can support palliative coordination by facilitating family and team communication and timely symptom-responsive interventions, with emphasis on preserving comfort, dignity, and ethical governance [6,20,43,61,66,109,110,170].

## Discussion

### Principal Findings

**Figure 4:**
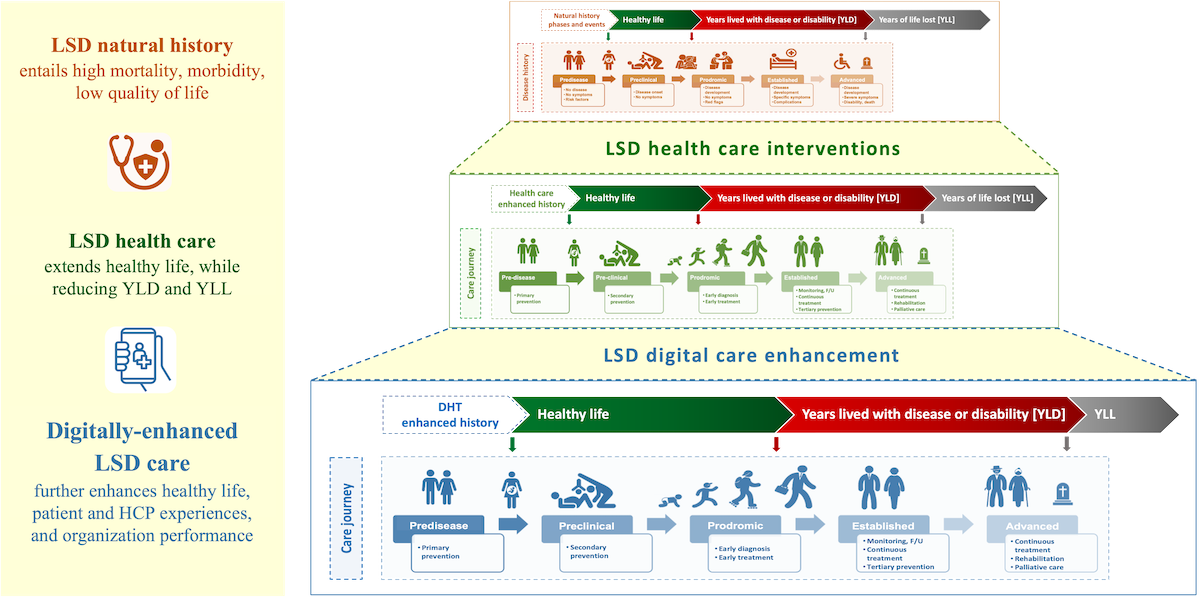
Towards a digitally enhanced care journey for lysosomal storage diseases. HCP: health care provider; LSD: lysosomal storage disorder.

This scoping review shows a rich research landscape on digital health applications for LSDs care, although the overall quality and maturity of the evidence remain limited. Most studied digital interventions cluster upstream along the care journey, focusing on enhancing diagnosis and early monitoring, with comparatively fewer applications in treatment intensification, rehabilitation, tertiary prevention, and end-of-life care. DHTs are used to shorten the road to diagnosis and to stabilize follow-up, and less to support care interventions, where disability, caregiver burden, and health system management tend to peak. In the context of the LSDs’ natural history and care journey phases (Figures 2 and 4), this pattern indicates that current evidence is more consistent on shortening diagnostic delays and optimizing surveillance, with a potential positive impact on years lived with disability and years of life lost.

Within this landscape, CC tools anchor real-world delivery (telemedicine, remote patient monitoring, ePRO, and registries), AI is used mainly for diagnostic decision support and risk stratification, and enabling informatics (omics, bioinformatics, non-AI imaging analyses) underpin discovery pipelines. Coverage is uneven across diseases: evidence is concentrated in FD and GD, with only small clusters for other LSDs and little or no activity for many of the 70+ additional entities (Table S2 in Multimedia Appendix 5). To avoid overgeneralization, we therefore synthesized findings stratified by LSD, labeled very low-count areas as signal-only or evidence gaps, and prioritized them in the Future Research section.

Across the evidence base, most outcomes relate to health care delivery performance, particularly technical diagnostic accuracy and workflow coordination. Technical accuracy alone represents over one-third of all outcome labels and nearly half of peer-reviewed reports, indicating that DHT evaluations in LSDs remain centered on proximal process measures (how well tools detect, classify, or transmit information) rather than on longer-term clinical, patient-centered, or societal outcomes. This pattern is typical of an emerging innovation field: early studies prioritize technical reliability and feasibility, while robust assessments of clinical benefit, quality of life, HCP and patient experience, cost-effectiveness, equity, or broader public-health impact are still limited.

These patterns must be methodologically interpreted also within the constraints of the underlying evidence profile. Although title and abstract screening was primarily conducted by one reviewer (AMCS), we used calibration and second-reviewer (LN) audits; residual selection error remains possible. This review was explicitly descriptive and aimed to map what has been studied rather than to infer effectiveness. Across included records, signals for AI (eg, diagnostic support, early detection, and risk stratification) and CC (eg, televisits, teleconsultations, remote follow-up, and monitoring) are emerging, but the predominance of small, single-center, observational designs and the presence of gray literature limit generalizability and constrain inference. Consistent with the scoping-review methodology, we did not conduct a formal risk-of-bias assessment. Instead, we charted study-level features and caveats descriptively and used them to temper interpretation. We accordingly avoided causal language and treated findings as hypothesis-generating and as implementation cues to inform future, more robust evaluations (eg, multisite studies using transparent datasets, predefined comparators, and standardized outcome measures). Our interpretations prioritized peer-reviewed evidence, while policy and strategy documents were used as contextual background only; we did not advance causal or policy claims that were not supported by study design and reporting quality. Additional constraints include language and time-window limits and potential publication and selection biases typical of rapidly evolving digital scenarios.

Taken together, these outcome- and phase-level patterns delineate where evidence is accumulating versus where it is still largely hypothetical. They highlight a need for future evaluations that (1) move beyond accuracy and process metrics toward robust clinical efficacy measures, as well as patient-centered, system-level, and societal endpoints; (2) include later care-journey phases such as rehabilitation, transition to adulthood, and palliative care; and (3) explicitly measure equity, safety, and cost implications. The subsequent Discussion subsections on challenges, counterevidence, and future research interpret these gaps as priorities for staged, theory-informed evaluation rather than as a mandate for immediate large-scale implementation.

### Challenges, Risks, and Biases

The evidence base also includes null or inconclusive findings, such as CC applications that did not improve adherence or use due to alert fatigue, workflow misalignment, or connectivity limitations; engagement platforms showing low sustained uptake; and AI models whose performance declined outside the derivation site or failed to outperform simpler baselines in small, heterogeneous cohorts [21,22,38,103,108].

Overall, Figures 2 and 4 suggest a functional split: AI is most often applied in early phases (screening, diagnosis, phenotyping), while CC supports continuity (follow-up, coordination, long-term monitoring). Given the largely descriptive evidence, deployment should be coupled with prospective, multisite, and equity-aware evaluations and transparent reporting [21,38,69,70].

AI-related risks reflect rare-disease data constraints: small single-center cohorts, incomplete or heterogeneous EHR data, missingness, and limited external validation drive overfitting and bias risk [21,38,69,70]. Proposed mitigation strategies include cross-registry pooling under findable, accessible, interoperable, and reusable principles, transfer learning, data augmentation or synthetic data, and routine auditing for bias and performance drift, aligned with emerging governance expectations (eg, EU AI Act) [103,164,171-173]. For CC deployments, the dominant concerns are interoperability with HIS and EHR, and equity (digital literacy, device, and connectivity access), such that CC can either bridge or widen the digital divide depending on implementation design [20-22,38,40,103,108,134,162,163].

Across included records, recurrent barriers to DHT scale-up in LSDs include infrastructure constraints, limited alignment across regulatory, HTA, and reimbursement requirements, and workforce skill gaps [38,128]. Interoperability, provider training, and robust privacy and security governance are repeatedly positioned as prerequisites, alongside patient trust, and clear accountability for data use [14,21,69,102].

### Future Research Priorities

Given rapid innovation and evolving governance, multistakeholder consensus processes (eg, consensus conferences informed by PICO-structured evidence syntheses) can help translate emerging evidence into fit-for-purpose recommendations for development, adoption, and evaluation of AI and CC in LSD care and in RDs more broadly [35,36].

Most included evidence is nonrandomized, single-center, with small samples and limited follow-up, constraining causal inference and generalizability [37,71,72,80]. Priorities, therefore, include standardized outcome sets, interoperable data capture, and multisite designs (including pragmatic and, where feasible, randomized evaluations) that can assess longer-term effectiveness, safety, equity, and implementation outcomes [28,29,35,72,79,80]. In rare, geographically dispersed LSD populations, DHTs may need to act both as interventions and as trial enablers (supporting decentralized recruitment, continuous outcome capture, and longitudinal follow-up) [28,79].

### Conclusions

This PRISMA-ScR–conformant review provides, to the authors’ knowledge, the first comprehensive mapping of DHT applications in LSDs care, spanning disease populations, intervention classes, outcome domains, and care-journey phases. Across 245 records, research activity clustered around a subset of LSDs, particularly FD and GD, and was concentrated in earlier care-journey phases such as screening, diagnosis, and early monitoring, with markedly fewer applications reported in treatment intensification, rehabilitation, transition, or end-of-life care. Care-delivery outcomes (including diagnostic accuracy, workflow coordination, and access to specialist expertise) dominated reporting, whereas patient-centered and societal outcomes remained comparatively scarce. Figure 4 synthesizes how these digitally enhanced activities align with LSDs’ natural history and conventional care pathways [24,102].

However, current evidence is insufficient to infer effectiveness or justify routine implementation. Across AI, CC, and other DHTs, most studies were small, single-center, observational, and exploratory, with only a modest contribution from gray literature. Consistent with the aims of a scoping review, these findings should be interpreted as descriptive and hypothesis-generating. Our mapping is intended to characterize patterns and identify gaps, not to support causal claims or comparative-effectiveness judgments.

Considering these findings, near-term priorities include stronger evidence generation (through RCTs, high-quality real-world evidence studies, and pragmatic, theory-informed implementation trials of AI and CC technologies), using transparent datasets and standardized outcome sets aligned with established evidence standards. Given that the current evidence base is appreciable in size, but heterogeneous and largely observational, expert consensus processes are needed to translate emerging signals into appropriate, practice-relevant guidance and to define minimum requirements for safe and meaningful clinical use. Policy integration and cross-border collaboration are not conclusions drawn from the current data but represent necessary enabling conditions for high-quality data availability in RDs, harmonized governance, interoperability, and future evidence generation. The 4-axis mapping framework and care-journey perspective presented here may help guide the design of these future evaluations and consensus efforts for LSDs and, where appropriate, other RDs.

## Appendix

Multimedia Appendix 1 The 22-item PRISMA-ScR checklist for scoping reviews.

Multimedia Appendix 2 Reproducibility logs of the scoping review search queries.

Multimedia Appendix 3 Examples of AI-driven and Connected Care (CC) Digital Health Technology (DHT) applications to enhance Lysosomal Storage Disease care pathways across all stages.

Multimedia Appendix 4 Examples of challenges for systemic integration of AI-driven and Connected Care (CC) Digital Health Technologies (DHTs).

Multimedia Appendix 5 Comparative and distribution evidence charting tables across LSD population, digital health interventions, patient-healthcare-societal outcomes, and care journey phases.

## Abbreviations

AI: artificial intelligence
CC: connected care
DHT: digital health technology
DL: deep learning
DMD: digital medical device
DMF: digital microfluidics
DSS: decision support system
DTx: digital therapeutics
EHR: electronic health record
ePRO: electronic patient-reported outcome
ERT: enzyme replacement therapy
EU: European Union
FD: Fabry disease
GD: Gaucher disease
GM1G: GM1 Gangliosidosis
GM2G: GM2 Gangliosidosis
HCP: health care provider
HIS: health information systems
HTA: health technology assessment
LLM: large language model
LSD: lysosomal storage disorder
mHealth: mobile health
ML: machine learning
MRI: magnetic resonance imaging
MS/MS: tandem mass spectrometry
NGS: next-generation sequencing
NLP: natural language processing
PCC: population, concept, context
PD: Pompe disease
PICO: Population, Intervention, Comparison, and Outcome
PRISMA-ScR: Preferred Reporting Items for Systematic Reviews and Meta-Analyses extension for Scoping Reviews
RCT: randomized controlled trial
RD: rare disease
VR: virtual reality
