# Supplementary material for: Artificial Intelligence, Connected Care, and Enabling Digital Health Technologies in Rare Diseases With a Focus on Lysosomal Storage Disorders: Scoping Review
Source: J Med Internet Res. 2026 Apr 2;28:e73612. doi: 10.2196/73612 (PMC13087560; doi:10.2196/73612)
Supplement: Multimedia Appendix 2 [file jmir_v28i1e73612_app2.docx]

# Reproducibility logs of the scoping review search queries

This appendix provides the full, stepwise, database-by-database search reproducibility logs for this scoping review, including the exact search strings (as entered), run dates, filters, and retrieved record counts for each source.

- Searches were executed on **September 30, 2024** and are reported here as a time-stamped snapshot to support transparent replication and auditability.
- For PubMed/MEDLINE, we report both the **baseline strategy** and a **sensitivity-expanded strategy**, with the latter operationalized by consolidating >80 DHT intervention terms identified during preliminary mapping into **55 Title/Abstract keywords** (reported in the stepwise log and incorporated into the final search string; see **#5** and **#10**).
- For Google Scholar, we report the query and operational procedures recognizing inherent reproducibility limitations (dynamic indexing and relevance ranking); therefore, we applied a predefined **stopping rule** by screening only the **first 1000 results sorted by relevance**, and we document the observed approximate yield displayed by the platform at the run date.
- AI-assisted discovery tools (eg, Consensus, SciSpace, Connected Papers) were used to **support identification** (including grey literature and citation-chaining) but did not replace eligibility assessment, data extraction, or synthesis; their outputs are logged for transparency while acknowledging that results may vary over time due to platform updates. All final inclusion decisions were made exclusively by human reviewers.

## Table S1. PUBMED / MEDLINE search

| **#** | **PubMed / MEDLINE inputs**  **(exact query string & filters)** | **Publication Period**  **(YYYY/MM/DD)** | **Records retrieved (n)** |
| --- | --- | --- | --- |
| 1 | *("Lysosomal Storage"[All Fields])* | Till 2024/09/30 | 4,655 |
| 2 | #1 AND *(2015/10/31:2024/9/30[pdat])* | From 2015/10/01  To 2024/09/30 | 1,113 |
| 3 | *("Digital"[Title/Abstract] OR "Digital Technology"[MeSH Terms] OR "Information Science"[MeSH Terms])* | Till 2024/09/30 | 3,997,722 |
| 4 | #3 AND *(2015/10/31:2024/9/30[pdat])* | From 2015/10/01  To 2024/09/30 | 1,207,674 |
| 5* | *("Augmented Reality (AR)"[Title/Abstract] OR "Biomedical Imaging"[Title/Abstract] OR "Clinical Decision Support"[Title/Abstract] OR "Digital Twins"[Title/Abstract] OR "Genomics"[Title/Abstract] OR "3D Printing"[Title/Abstract] OR "Pharmacogenomics"[Title/Abstract] OR "Precision Medicine"[Title/Abstract] OR "Virtual Reality (VR)"[Title/Abstract] OR "Algorithm"[Title/Abstract] OR "Artificial Intelligence"[Title/Abstract] OR "Blockchain"[Title/Abstract] OR "Cloud"[Title/Abstract] OR "Cognitive Computing"[Title/Abstract] OR "Computer Vision"[Title/Abstract] OR "Cybersecurity"[Title/Abstract] OR "Data Mining"[Title/Abstract] OR "DeepGestalt"[Title/Abstract] OR "Digital Health"[Title/Abstract] OR "Digital Health Platforms"[Title/Abstract] OR "E-Health"[Title/Abstract] OR "Electronic Health Records"[Title/Abstract] OR "Expert Systems"[Title/Abstract] OR "Facial analysis"[Title/Abstract] OR "Fuzzy Logic"[Title/Abstract] OR "Genetic Algorithms"[Title/Abstract] OR "Health Data Analytics"[Title/Abstract] OR "Health Information System"[Title/Abstract] OR "Health Apps"[Title/Abstract] OR "Image Recognition"[Title/Abstract] OR "IoT (Internet of Things)"[Title/Abstract] OR "Knowledge Representation"[Title/Abstract] OR "Machine Learning"[Title/Abstract] OR "mHealth"[Title/Abstract] OR "Mobile"[Title/Abstract] OR "Mobile App"[Title/Abstract] OR "Natural Language Processing"[Title/Abstract] OR "Network"[Title/Abstract] OR "Online"[Title/Abstract] OR "Patient Monitoring"[Title/Abstract] OR "Patient Portals"[Title/Abstract] OR "Predictive Analytics"[Title/Abstract] OR "Remote Patient Monitoring"[Title/Abstract] OR "Robotics"[Title/Abstract] OR "Sentiment Analysis"[Title/Abstract] OR "Sensor"[Title/Abstract] OR "Smartphone"[Title/Abstract] OR "Social Media"[Title/Abstract] OR "Speech Recognition"[Title/Abstract] OR "Swarm Intelligence"[Title/Abstract] OR "Telehealth"[Title/Abstract] OR "Telehealth Services"[Title/Abstract] OR "Telemedicine"[Title/Abstract] OR "Virtual Care"[Title/Abstract] OR "Wearable"[Title/Abstract])* | Till 2024/09/30 | 2,097,869 |
| 6 | #5 AND *(2015/10/31:2024/9/30[pdat])* | From 2015/10/01  To 2024/09/30 | 1,214,314 |
| 7 | #1 AND #3 | Till 2024/09/30 | 783 |
| 8 | #1 AND #3 AND *(2015/10/31:2024/9/30[pdat])* | From 2015/10/01  To 2024/09/30 | 218 |
| 9 | #1 AND (#3 OR #5) | Till 2024/09/30 | 1,113 |
| **10** | **#1 AND (#3 OR #5) AND *(2015/10/31:2024/9/30[pdat])*** | **From 2015/10/01**  **To 2024/09/30** | **408** |

* This sensitivity expansion consolidates >80 DHT intervention terms observed during preliminary mapping into **55 Title/Abstract keywords**, to increase recall without broadening the LSD concept.

## Table S2. GOOGLE SCHOLAR search

| **#** | **Google Scholar inputs**  **(exact query string & filters)** | **Publication Date**  **(YYYY/MM/DD)** | **Records retrieved (n)** |
| --- | --- | --- | --- |
| 11 | *"Lysosomal Storage"* | Till 2024 | About 48,000 |
| 12 | #11 AND (Filter:2015-2024) | From 2015 to 2024 | About 21,200 |
| 13 | *"Digital Technology" OR "Information Science"* | Till 2024 | About 17,800 |
| 14 | #13 AND (Filter:2015-2024) | From 2015 to 2024 | About 14,900 |
| 15 | *("Augmented Reality" OR "Biomedical Imaging" OR "Clinical Decision Support" OR "Digital Twins" OR "Genomics" OR "3D Printing" OR "Pharmacogenomics" OR "Precision Medicine" OR "Virtual Reality" OR "Algorithm" OR "Artificial Intelligence" OR "Blockchain" OR "Cloud" OR "Cognitive Computing" OR "Computer Vision" OR "Cybersecurity" OR "Data Mining" OR "DeepGestalt" OR "Digital Health" OR "Digital Health Platforms" OR "E-Health" OR "Electronic Health Records" OR "Expert Systems" OR "Facial analysis" OR "Fuzzy Logic" OR "Genetic Algorithms" OR "Health Data Analytics" OR "Health Information System" OR "Health Apps" OR "Image Recognition" OR "IoT" OR "Internet of Things" OR "Knowledge Representation" OR "Machine Learning" OR "mHealth" OR "Mobile" OR "Mobile App" OR "Natural Language Processing" OR "Network" OR "Online" OR "Patient Monitoring" OR "Patient Portals" OR "Predictive Analytics" OR "Remote Patient Monitoring" OR "Robotics" OR "Sentiment Analysis" OR "Sensor" OR "Smartphone" OR "Social Media" OR "Speech Recognition" OR "Swarm Intelligence" OR "Telehealth" OR "Telehealth Services" OR "Telemedicine" OR "Virtual Care" OR "Wearable")* | Till 2024 | About 771,000 |
| 16 | #15 AND (Filter:2015-2024) | From 2015 to 2024 | About 633,000 |
| 17 | #11 AND #13 | Till 2024 | About 1,040 |
| 18 | #11 AND #13 AND (Filter:2015-2024) | From 2015 to 2024 | About 755 |
| 19 | #11 AND (#13 OR #15) | Till 2024 | About 18,500 |
| 20 | #11 AND (#13 OR #15) AND (Filter:2015-2024) | From 2015 to 2024 | About 15,300 |
| 21 | *"telemedicine" OR "artificial intelligence"* | Till 2024 | About 156,000 |
| 22 | #21 AND (Filter:2015-2024) | From 2015 to 2024 | About 76,400 |
| 23 | #11 AND (#13 OR #21) | Till 2024 | About 7,020 |
| 24 | #11 AND (#13 OR #21) AND (Filter:2015-2024) | From 2015 to 2024 | About 4,070 |
| **25*** | **#24 with Stopping Rule (1,000 max retrieved records, ordered by Relevance)** | **From 2015 to 2024** | **1,000** |

* Counts are approximate as displayed by Google Scholar; relevance ranking is dynamic; we therefore operationalized a pre-specified stopping rule and documented the run date.

## Table S3. CONSENSUS AI-assisted search

| **#** | **Consensus.app inputs**  **(exact query string & filters)** | **Publication Date**  **(YYYY/MM/DD)** | **Records retrieved (n)** |
| --- | --- | --- | --- |
| 33 | *“Digital Health for Rare Diseases: A Scoping Review of Artificial Intelligence and Connected Care in Lysosomal Storage Disorders” [Filters: Deep Search; Medicine, Biology, Psychology, Social Science, Computer Science databases; 2015-2024]* | From 2015 to 2024 | 1,130 |
| **34** | **#33 with Stopping Rule (50 max retrieved records, ordered by relevance after automated screening and inclusion)** | **From 2015 to 2024** | **50** |

## Table S4. SCISPACE AI-assisted search

| **#** | **SciSpace.com inputs**  **(exact query string & filters)** | **Publication Date**  **(YYYY/MM/DD)** | **Records retrieved (n)** |
| --- | --- | --- | --- |
| **35** | ***“Digital Health for Rare Diseases: A Scoping Review of Artificial Intelligence and Connected Care in Lysosomal Storage Disorders” [Filters: Deep Search; Biomedical databases; published from Oct 31, 2015 to Sept 30, 2024]*** | **From 2015/10/01**  **to 2024/09/30** | **163** |
| 36 | Semi-automated screening, after prompt to select the 10 top relevance papers out of #35, to be reviewed and approved by humans, to trigger Connected Papers prior and derivative mapping (see below). | From 2015/10/01  to 2024/09/30 | 10 |

## Table S5. CONNECTED PAPERS AI-assisted search

| **#** | **Connectedpapers.com inputs**  **(DOI)** | **Publication Date**  **(YYYY/MM/DD)** | **Records retrieved (n)**  **[Prior + Derivative]** |
| --- | --- | --- | --- |
| 37 | <https://doi.org/10.3390/jcm13113325> | From 2015 to 2024 | 10 + 4 |
| 38 | <https://doi.org/10.1016/j.jclinepi.2024.111517> | From 2015 to 2024 | 5 + 4 |
| 39 | <https://doi.org/10.3389/fneur.2023.1108222> | From 2015 to 2024 | 1 + 7 |
| 40 | <https://doi.org/10.1186/s13023-019-1182-6> | From 2015 to 2024 | 2 + 8 |
| 41 | <https://doi.org/10.1186/s13023-023-02999-6> | From 2015 to 2024 | 7 + 0 |
| 42 | <https://doi.org/10.1186/s13023-020-01473-x> | From 2015 to 2024 | 2 + 8 |
| 43 | <https://doi.org/10.1016/j.bone.2024.117142> | From 2015 to 2024 | 2 + 6 |
| 44 | <https://doi.org/10.1002/hbm.26599> | From 2015 to 2024 | 8 + 6 |
| 45 | <https://doi.org/10.1038/S41746-019-0143-8> | From 2015 to 2024 | 0 + 4 |
| 46 | <https://doi.org/10.4274/jpr.galenos.2022.04206> | From 2015 to 2024 | 8 + 10 |
| **47** | **#37+#38+#39+#40+#4+#42+#43+#44+#45+#46** | **From 2015 to 2024** | **102** |

## Table S6. CLINICALTRIALS.GOV search

| **#** | **Clinicaltrials.gov inputs**  **(exact query string & filters)** | **Publication Date**  **(YYYY/MM/DD)** | **Records retrieved (n)** |
| --- | --- | --- | --- |
| 26 | *(AREA[ConditionSearch](Lysosomal Storage Disorders)*  *OR AREA[BasicSearch](Lysosomal Storage Diseases))*  *AND AREA[StartDate] RANGE[2015-10-31,2024-09-30]*  *AND AREA[StdAge](CHILD OR ADULT OR OLDER_ADULT)* | From 2015/10/01  to 2024/09/30 | 479 |
| 27 | *#26 AND AREA[InterventionSearch](digital)* | From 2015/10/01  to 2024/09/30 | 5 |
| 28 | *#26 AND AREA[InterventionSearch](artificial intelligence)* | From 2015/10/01  to 2024/09/30 | 2 |
| 29 | *#26 AND AREA[InterventionSearch](telemedicine)* | From 2015/10/01  to 2024/09/30 | 2 |
| 30 | *#26 AND AREA[InterventionSearch](electronic)* | From 2015/10/01  to 2024/09/30 | 5 |
| 31 | *#26 AND AREA[InterventionSearch](mobile)* | From 2015/10/01  to 2024/09/30 | 1 |
| **32** | ***#26 AND (#27 OR #28 OR #29 OR #30 OR #31)*** | **From 2015/10/01**  **to 2024/09/30** | **14** |

## Table S7. Summary of main and complementary searches (pre-screening, retrieved records)

| **#** | **Search Tool** | **Data Source** | **Inputs**  **(strings, filters, DOI)** | **Period searched**  **[yyyy/mm/dd]** | **Records retrieved (n)** |
| --- | --- | --- | --- | --- | --- |
| a | **PubMed/MEDLINE** | Core Database | #10 | From 2015/10/01  to 2024/09/30 | 408 |
| b | **Google Scholar** | Complementary Database | #25 | From 2015/10/01  to 2024/09/30 | 1,000 |
| c | **Consensus.app** | Complementary Databases | #34 | From 2015/10/01  to 2024/09/30 | 50 |
| d | **SciSpace.com** | Complementary Databases | #35 | From 2015/10/01  to 2024/09/30 | 163 |
| e | **ConnectedPapers.com** | Complementary Databases | #47 | From 2015/10/01  to 2024/09/30 | 102 |
| f | **ClinicalTrials.gov** | Registry | #32 | From 2015/10/01  to 2024/09/30 | 14 |
| g | **Others** | Citations, web, authors’ suggestions | N/A | From 2015/10/01  to 2024/09/30 | 14 |
| h | **TOTAL** | **[Cumulative]** | **[Cumulative]** | **From 2015/10/01**  **to 2024/09/30** | **1,751** |
