# Supplementary material for: Artificial Intelligence, Connected Care, and Enabling Digital Health Technologies in Rare Diseases With a Focus on Lysosomal Storage Disorders: Scoping Review
Source: J Med Internet Res. 2026 Apr 2;28:e73612. doi: 10.2196/73612 (PMC13087560; doi:10.2196/73612)
Supplement: Multimedia Appendix 3 [file jmir_v28i1e73612_app3.docx]

## **Examples of AI-driven and Connected Care (CC) Digital Health Technology (DHT) applications to enhance Lysosomal Storage Disease (LSD) care pathways across all stages.**

| **PRIMARY**  **PREVENTION** | - **AI-driven genomic analysis platforms** are pivotal in primary prevention by identifying individuals at risk for LSDs. - **Pre-conception interventions,** including family genetic counseling and planning delivered via **tele-genetics platforms,** empower patients and families to understand inheritance patterns, lifestyle changes, and other proactive health measures. |
| --- | --- |
| **SECONDARY PREVENTION**  **& DIAGNOSIS** | - **Digital microfluidics** and **AI-driven diagnostics** for early detection of disease markers, notably in neonatal screening, enabling earlier detection and potentially improving long-term outcomes. - **AI-driven diagnostics** utilize **facial recognition software** and **genomic analysis** platforms for early detection of diseases - **AI-DHTs** based on **Machine Learning and Deep Learning algorithms,** analyze **diagnostic images** to detect critical biomarkers and structural anomalies, providing essential insights for personalized treatments. - **Natural Language Processing** (NLP) and **Large Language Models** (LLMs) extract information from EHRs and unstructured data sources, enabling **AI-powered Clinical Decision Support Systems (CDSS).** They can facilitate identification of disease-specific LSD patterns and support diagnostic and treatment decisions for LSDs, particularly in case of limited clinical expertise or unclear symptoms. - **Telemedicine** transforms the diagnostic landscape by facilitating HCP-patient **televisits** and enhancing HCP-HCP collaboration through **teleconsultations.** - **Next Generation Sequencing (NGS)** advances **genetic analysis**, identifying mutations and enabling precision medicine. By providing detailed genetic profiles NGS enhances the speed and accuracy of diagnosis - Collectively, these **DHTs** enhance timely diagnosis, diagnostic accuracy and expedite appropriate, personalized and multidisciplinary interventions, leading to improved health outcomes and patient satisfaction. |
| **TREATMENT** | - **AI-driven drug development** tools use **genetic, proteomic, and metabolomic** data to tailor therapies, improving treatment outcomes and reducing adverse effects. - **HCP-Patient tele-visits,** including **tele-genetics**, ensure timely and frequent updates on treatment, promoting accurate and prompt initiation of personalized therapies. - **HCP-HCP tele-consultations** facilitate access to expert second opinions, enhance coordinated and multidisciplinary care, and optimize treatment decisions, especially for complex cases. - **AI-driven and CC DHTs** can enhance precision and responsiveness of treatments, ensuring patients receive optimal care tailored to their specific needs, ultimately leading to better health outcomes. - **Digital Therapeutics (DTx)** could enable **digitally-delivered treatment** for common LSD symptoms and comorbidities, in particular psychological symptoms, such as depression and sleeping disorders. |
| **MONITORING & FOLLOW-UP** | - **Remote Patient Monitoring (RPM) Digital Medical Devices (DMDs)** provide tracking of vital signs and symptoms, treatment efficacy, and disease progression, enabling HCPs to make timely and personalized adjustments to treatment protocols. - **Telemedicine** platforms enhance monitoring and care capabilities by allowing for **recurrent remote tele-visits, HCP-HCP teleconsultations** and **remote monitoring**, crucial for managing chronic conditions like cardiovascular complications in FD or skeletal issues in GD. This ensures patients receive consistent care, optimizing the need for hospital visits. - **Digital Patient Support Programs** and **DMDs**, including **ePRO monitoring, wearables** and **mHealth apps,** enhance patient self-management, monitoring, dynamic diagnostic adjustments and treatment optimization, maintaining ongoing surveillance. - **Structured real-world data** from reported symptoms or connected devices may be combined within **HISs and EHRs,** offering a holistic view of the patient's health status, and enabling collaboration on treatment plans, streamlining the care process and improving decision-making. - **Digital patient communities** significantly enrich the patient experience by providing flexible access to healthcare services and educational content. |
| **REHABILI- TATION** | - **DHTs** like **DMDs** and **DTx** can enable at-home rehabilitation therapy, enhancing physical and psychological recovery. - **Tele-rehabilitation** platforms further support consistent, personalized therapy sessions. |
| **TERTIARY**  **PREVENTION** | - **AI-driven** **CDSS** are pivotal in managing existing conditions, mitigating progression, or preventing complications. They can support dynamic adjustments to treatment protocols based on real-world monitoring of disease markers, risk of disease progression and of comorbidities development. - **DMDs** for remote monitoring**, HCP-Patient televisits** complementing regular follow-ups and **multidisciplinary HCP-HCP** **teleconsultations** can also enhance tertiary prevention. |
| **END-OF-LIFE CARE** | - **Telemedicine** facilitates crucial communication between families and healthcare teams, while **DMDs for Remote Patient Monitoring** track vital signs and symptoms for **timely medical interventions.** - **DHTs** enhance patient comfort and dignity through personalized symptom management and emotional support, ensuring treatments are adapted to patient needs. |
