# Supplementary material for: Artificial Intelligence, Connected Care, and Enabling Digital Health Technologies in Rare Diseases With a Focus on Lysosomal Storage Disorders: Scoping Review
Source: J Med Internet Res. 2026 Apr 2;28:e73612. doi: 10.2196/73612 (PMC13087560; doi:10.2196/73612)
Supplement: Multimedia Appendix 4 [file jmir_v28i1e73612_app4.docx]

# **Challenges for systemic integration of AI-driven and Connected Care (CC) Digital Health Technologies (DHTs).**

| **ACROSS ALL CARE JOURNEY PHASES** | - **Systemic integration** of AI-driven and CC DHTs requires **adequate technology infrastructure**, **data availability**, data **interoperability**, EHR and workflow **integration**, data **privacy** and **security** compliance. |
| --- | --- |
|  | - **Ethical Considerations** raise discussions around **privacy** and the risk for **genetic discrimination**, requiring careful management of **patient data** and prevention of **biases** inferred by AI algorithms. |
|  | - **Fit-for-purpose health technology assessment** standards and **policies** for **equitable access** and **appropriate adoption** of **safe and** effective **DHTs** are also needed. |
|  | - **Capacity-building and continuous training** are essential for HCP, caregivers and patients to ensure effective use and **integration** into daily healthcare routines. |
|  | - Process and **workflow** innovation is essential. |
|  | - Ongoing **adaptation** to technology advancements and software updates is to be accounted for. |
|  | - **Effective integration** into clinical settings and **validation for** effectiveness are essential to ensure such DHTs meet security, safety, usability and clinical **standards** and enhance patient care. |
|  | - **Policy interventions** are needed to ensure technology is **equitably** accessible to all and HCPs should **balance** digital interactions with **essential human contact.** |
|  | - While these technologies elevate also the quality of end-life care, they require **thoughtful integration** to ensure treatments remain both **personalized and ethically sound.** |
