# Supplementary material for: Artificial Intelligence, Connected Care, and Enabling Digital Health Technologies in Rare Diseases With a Focus on Lysosomal Storage Disorders: Scoping Review
Source: J Med Internet Res. 2026 Apr 2;28:e73612. doi: 10.2196/73612 (PMC13087560; doi:10.2196/73612)
Supplement: Multimedia Appendix 5 [file jmir_v28i1e73612_app5.docx]

# Comparative and distribution evidence charting tables across LSD population, interventions, outcomes, and care journey phases

This appendix reports the full evidence charting tables extracted from the included studies. These tables support the descriptive mapping objectives of this scoping review by documenting study characteristics, LSD populations, digital health technology (DHT) categories (artificial intelligence, connected care, and other enabling DHTs), care-journey phases, and reported outcomes.

All tables are directly referenced in the Results section and are fully consistent with the eligibility criteria, study counts, and categorizations reported in the manuscript and in Multimedia Appendix 2 (search reproducibility logs).

Totals/subtotals marked with * may not equal the sum of sub-rows because single papers can carry multiple labels; label-column sums are always exact. Total or denominator for Labeled Papers is “245”, for Labeled Peer Reviewed (PR) Papers is “226”.

## Table S1. Disease coverage by DHT intervention class

Rows list LSDs. Columns show AI/CC/other-DHT “Labels”, “Row Total Labels”, “Labeled Papers”, “Labeled PR-Papers”, and “% of All PR-Papers”.

| **Code** | **LSD Population** | **AI Labels** | **CC Labels** | **Other DHTs Labels** | **Row**  **Total Labels** | **% of**  **All Labels** | **Labeled Papers** | **Labeled**  **PR-Papers** | **% of All**  **PR-Papers** |
| --- | --- | --- | --- | --- | --- | --- | --- | --- | --- |
| D.1 | Fabry Disease | **16** | **38** | 46 | 100 | 26.0% | 77 | 71 | **31.4%** |
| D.2 | Gaucher Disease | **14** | **29** | 41 | 84 | 21.8% | 64 | 59 | **26.1%** |
| D.3 | Mucopolysaccharidosis | **3** | **17** | 36 | 56 | 14.5% | 50 | 47 | **20.8%** |
| D.4 | Pompe Disease | **4** | **23** | 28 | 55 | 14.3% | 44 | 41 | **18.1%** |
| D.5 | Niemann-Pick | **1** | **12** | 28 | 41 | 10.6% | 32 | 31 | **13.7%** |
| D.6 | Krabbe Disease | **0** | **2** | 10 | 12 | 3.1% | 11 | 11 | **4.9%** |
| D.7 | Metachromatic Leukodystrophy | **1** | **4** | 8 | 13 | 3.4% | 9 | 9 | **4.0%** |
| D.8 | Gangliosidosis | **1** | **3** | 7 | 11 | 2.9% | 8 | 7 | **3.1%** |
| D.9 | Batten Disease | **1** | **3** | 5 | 9 | 2.3% | 7 | 7 | **3.1%** |
| D.10 | Aspartyl-glucosaminuria | **2** | **0** | 2 | 4 | 1.0% | 3 | 3 | **1.3%** |
| **D.Tot** | **All diseases** | **43** | **131** | **211** | **385** | **100.0%** | **245*** | **226*** | **100.0%*** |

##

## Table S2. Distribution of DHT intervention subtypes

Rows list AI, CC, and other DHT subtypes. Columns show “Labels”, “Labeled Papers”, “Labeled PR-Papers”, and “% of All PR-Papers”.

| **Code** | **Intervention subtype** | **Labels** | **% of All Labels** | **Labeled**  **Papers** | **Labeled**  **PR-Papers** | **% of All**  **PR-Papers** |
| --- | --- | --- | --- | --- | --- | --- |
| **T.1** | **AI DHTs** | **52** | **13.7%** | **45*** | **40*** | **17.7%*** |
| T.1.1 | AI-driven Risk Prediction and Patient Stratification | 4 | 1.1% | 4 | 4 | 1.8% |
| T.1.2 | AI-driven Clinical Diagnostics and Therapeutics | 41 | 10.8% | 41 | 39 | 17.3% |
| T.1.3 | AI-driven Research and Development (R&D) | 7 | 1.8% | 7 | 6 | 2.7% |
| **T.2** | **CC DHTs** | **122** | **32.1%** | **98*** | **89*** | **39.4%*** |
| T.2.1 | Telemedicine | 28 | 7.4% | 28 | 27 | 11.9% |
| T.2.2 | Digital Medical Devices (DMDs) | 53 | 13.9% | 37 | 34 | 15.0% |
| T.2.3 | Patient Engagement Tools | 10 | 2.6% | 10 | 7 | 3.1% |
| T.2.4 | Data Integration Systems | 31 | 8.2% | 31 | 31 | 13.7% |
| **T.3** | **Other ENABLING DHTs** | **206** | **54.2%** | **154*** | **144*** | **63.7%*** |
| T.3.1 | Bioinformatics pipelines | 55 | 14.5% | 55 | 50 | 22.1% |
| T.3.2 | “Omics” analysis | 53 | 13.9% | 53 | 48 | 21.2% |
| T.3.3 | Imaging analysis (non-AI) | 26 | 6.8% | 26 | 24 | 10.6% |
| T.3.4 | Computational modeling/simulation | 21 | 5.5% | 21 | 21 | 9.3% |
| T.3.5 | Digital microfluidics | 18 | 4.7% | 18 | 18 | 8.0% |
| T.3.6 | Next-generation sequencing (NGS) | 17 | 4.5% | 17 | 17 | 7.5% |
| T.3.7 | Precision-medicine | 13 | 3.4% | 13 | 12 | 5.3% |
| T.3.8 | VR/AR | 2 | 0.5% | 2 | 2 | 0.9% |
| T.3.9 | Trial simulation (non-AI) | 1 | 0.3% | 1 | 1 | 0.4% |
| **T.1+2+3** | **All subtypes** | **380** | **100.0%** | **245*** | **226*** | **100.0%*** |

##

## Table S3. Outcome domains by DHT intervention class

Rows group patient, healthcare-system, and societal outcomes. Columns show AI/CC/other-DHT “Labels”, “Row Total Labels”, “Labeled Papers”, “Labeled PR-Papers”, and “% of All PR-Papers”.

| **Code** | **Outcome Domain** | **AI Labels** | **CC Labels** | **Other DHTs Labels** | **Row**  **Total Labels** | **% of**  **All Labels** | **Labeled Papers** | **Labeled**  **PR-Papers** | **% of All**  **PR-Papers** |
| --- | --- | --- | --- | --- | --- | --- | --- | --- | --- |
| **O.1** | **Patient Outcomes** | **15** | **70** | **32** | **117** | **27.2%** | **74*** | **71*** | **31.4%*** |
| O.1.1 | Patient Safety | 3 | 8 | 6 | 17 | 4.0% | 10 | 10 | 4.4% |
| O.1.2 | Patient Privacy | 6 | 19 | 14 | 39 | 9.1% | 24 | 22 | 9.7% |
| O.1.3 | Patient Experience | 4 | 32 | 9 | 45 | 10.5% | 40 | 38 | 16.8% |
| O.1.4 | Patient Awareness and Education | 2 | 11 | 3 | 16 | 3.7% | 12 | 10 | 4.4% |
| **O.2** | **Healthcare Delivery Outcomes** | **52** | **84** | **143** | **279** | **64.9%** | **198*** | **191*** | **84.5%*** |
| O.2.1 | Technical Diagnostic Accuracy | 39 | 31 | 87 | 157 | 36.5% | 123 | 114 | 50.4% |
| O.2.2 | System and Data Security | 1 | 19 | 14 | 34 | 7.9% | 24 | 22 | 9.7% |
| O.2.3 | Clinical Efficacy | 6 | 5 | 18 | 29 | 6.7% | 24 | 21 | 9.3% |
| O.2.4 | Cost and Organizational Performance | 5 | 15 | 21 | 41 | 9.5% | 28 | 27 | 11.9% |
| O.2.5 | Healthcare Provider Experience | 0 | 10 | 1 | 11 | 2.6% | 10 | 9 | 4.0% |
| O.2.6 | Healthcare Provider Awareness and Education | 1 | 4 | 2 | 7 | 1.6% | 4 | 4 | 1.8% |
| **O.3** | **Societal Outcomes** | **4** | **12** | **18** | **34** | **7.9%** | **24*** | **24*** | **10.6%*** |
| O.3.1 | Public Health and Social Determinants of Health | 1 | 4 | 5 | 10 | 2.3% | 9 | 9 | 4.0% |
| O.3.2 | Regulatory and Ethical Compliance | 3 | 8 | 13 | 24 | 5.6% | 17 | 17 | 7.5% |
| **O.1+2+3** | **All outcomes** | **71** | **166** | **193** | **430** | **100.0%** | **245*** | **226*** | **100.0%*** |

##

## Table S4. DHT intervention class by care-journey phase

Rows show care journey phases. Columns show AI/CC/Other-DHT “Labels”, “Row Total Labels”, “Labeled Papers”, “Labeled PR-Papers”, and “% of All PR-Papers”.

| **Code** | **Care Journey Phase** | **AI Labels** | **CC Labels** | **Other DHTs Labels** | **Row**  **Total Labels** | **% of**  **All Labels** | **Labeled Papers** | **Labeled**  **PR-Papers** | **% of All**  **PR-Papers** |
| --- | --- | --- | --- | --- | --- | --- | --- | --- | --- |
| P.1 | Primary prevention | 2 | 6 | 3 | 11 | 2.3% | 7 | 6 | 2.7% |
| P.2 | Secondary prevention and diagnosis | 39 | 63 | 120 | 222 | 47.0% | 174 | 160 | 70.8% |
| P.3 | Treatment | 12 | 32 | 51 | 95 | 20.1% | 78 | 71 | 31.4% |
| P.4 | Monitoring and follow-up | 16 | 47 | 53 | 116 | 24.6% | 94 | 90 | 39.8% |
| P.5 | Rehabilitation | 2 | 6 | 5 | 13 | 2.8% | 9 | 8 | 3.5% |
| P.6 | Tertiary prevention | 2 | 4 | 3 | 9 | 1.9% | 5 | 5 | 2.2% |
| P.7 | End-of-life care | 0 | 4 | 2 | 6 | 1.3% | 5 | 5 | 2.2% |
| **P.Tot** | **All phases** | **73** | **162** | **237** | **472** | **100.0%** | **245*** | **226*** | **100.0%*** |
